# Safety and feasibility of allogeneic cord blood-derived cell therapy in preterm infants with severe brain injury (ALLO trial): a phase-1 trial protocol

**DOI:** 10.1136/bmjopen-2025-100389

**Published:** 2025-06-18

**Authors:** Abdul Razak, Kristyn Connelly, Rod W Hunt, Suzanne L Miller, Courtney A McDonald, Graham Jenkin, Lindsay Zhou, Madison CB Paton, Michelle Martin, Lin Liu, Cathie Hart, Ngaire J Elwood, Atul Malhotra

**Affiliations:** 1 Department of Paediatrics, Monash University, Melbourne, Victoria, Australia; 2 Monash Newborn, Monash Children’s Hospital, Melbourne, Victoria, Australia; 3 The Ritchie Centre, Hudson Institute of Medical Research, Melbourne, Victoria, Australia; 4 Cerebral Palsy Alliance Research Institute, Cerebral Palsy Alliance, Sydney, New South Wales, Australia; 5 Department of Obstetrics and Gynaecology, Monash University, Melbourne, Victoria, Australia; 6 Department of Paediatric Haematology-Oncology, Monash Children’s Hospital, Melbourne, Victoria, Australia; 7 Murdoch Children’s Research Institute, The Royal Children's Hospital, Melbourne, Victoria, Australia; 8 BMDI Cord Blood Bank, The Royal Children's Hospital, Melbourne, Victoria, Australia; 9 Australian Red Cross Lifeblood, Melbourne, Victoria, Australia; 10 Department of Paediatrics, University of Melbourne, Melbourne, Victoria, Australia

**Keywords:** Clinical Trial, NEONATOLOGY, Brain Injuries, Cell biology, Developmental neurology & neurodisability

## Abstract

**ABSTRACT:**

**Introduction:**

Severe intraventricular haemorrhage (IVH) and white matter injury (WMI) are major neurological complications in preterm infants, leading to long-term neurodevelopmental impairments. Despite advances in neonatal care, effective treatments are lacking. Umbilical cord blood cell (UCBC) therapy shows neuroprotective potential, with autologous sources ideal but often not feasible due to the unpredictability of preterm births. Allogeneic UCBCs offer an alternative, although immunogenicity and human leucocyte antigen (HLA) compatibility present challenges with knowledge gaps in their relevance in neonatal populations. This study aims to assess the feasibility and safety of partially HLA-matched allogeneic UCBC therapy in preterm infants with severe brain injury.

**Methods:**

The ALLO trial is an open-label, phase I, single-arm feasibility and safety study conducted at Monash Children’s Hospital, Victoria, Australia. Preterm infants born before 28 weeks (ALLO-1) or between 28 weeks and 36+6 weeks (ALLO-2) gestational age with severe brain injury identified on neuroimaging will be enrolled. Severe brain injury is defined as grade 3 or 4 IVH or significant WMI. Exclusion criteria include major congenital anomalies or redirection to comfort care. Eligible infants will receive a single intravenous infusion of unrelated, allogeneic, partially HLA-matched (4/6 or 5/6 HLA match) UCBCs sourced from a public cord blood bank. The target dose is 50 million total nucleated cells per kilogram body weight. Infusion will occur within 2–3 weeks of confirmation of eligibility, contingent on clinical stability and absence of active sepsis. Primary outcome includes: (1) feasibility, defined as having more than 60% of enrolled infants with an eligible allogeneic partially matched cord blood unit available and (2) safety, defined as absence of severe adverse events within 48 hours of infusion or graft-versus-host disease within 3 months of infusion. Secondary outcomes include survival, neonatal morbidities, neurodevelopmental assessments and serum cytokine analysis.

**Ethics and dissemination:**

Monash HREC has granted full ethics approval (RES-23-0000-297A) for the study, including the research use of allogeneic cord blood from compassionate donations by healthy donors, facilitated through the Bone Marrow Donor Institute Cord Blood Bank within the AusCord network. Findings will be disseminated through peer-reviewed publications and conference presentations, contributing to the development of novel neuroreparative therapies for preterm brain injury.

**Trial registration number:**

ACTRN12623001352695 (The Australian New Zealand Clinical Trials Registry).

**STRENGTHS AND LIMITATIONS OF THIS STUDY**

- The study evaluates allogeneic umbilical cord blood cell (UCBC) therapy as a potential neuroreparative treatment for preterm infants with severe brain injury, addressing the limitation of autologous UCBC availability and offering hope for infants facing life-threatening or disabling outcomes.
- The trial is primarily designed for safety, with active risk mitigation and ongoing monitoring of infusional and other immune toxicity.
- The study explores the feasibility of using UCBCs sourced from a public cord blood bank for neonatal applications, creating pathways for future clinical trials using unrelated allogeneic UCBCs.
- While this is a world-first study of allogeneic UCBC in preterm infants, once feasibility and safety are established, larger trials will be needed in the future to investigate efficacy.

## Introduction

 Severe intraventricular haemorrhage (IVH) and white matter injury (WMI) represent significant neurological morbidities in preterm infants, contributing substantially to long-term neurodevelopmental impairments. Despite advances in neonatal care, there remain few effective interventions^1^ to prevent or reduce severe IVH and WMI, and their incidence has shown little improvement over the past decade.^2^ Recent data from the Australian and New Zealand Neonatal Network reveal that severe IVH affects approximately 5% of infants born before 32 weeks of gestation and 11.6% of those born before 27 weeks.^3^ These conditions are associated with a high risk of adverse neurodevelopmental outcomes, with no significant change in recent trends over time.^2^ Notably, up to 65% of survivors with severe IVH or WMI develop cerebral palsy.^2^ Given the ongoing burden despite preventative efforts, it is essential to focus on developing restorative therapies to improve outcomes for this preterm cohort.

Treatment options for mitigating these sequelae remain limited,^1^ underscoring an urgent need for innovative neuroreparative therapies to improve outcomes for this vulnerable population.

Umbilical cord blood derived cell (UCBC) therapy has emerged as a promising therapeutic option for neonatal brain injuries due to its anti-inflammatory, neuroprotective and regenerative properties.^4^,^11^ Autologous UCBC, leveraging a baby’s own cord blood, is a feasible option, when available, as demonstrated in our recent CORD-SaFe study.^12 13^ However, logistical challenges, such as precipitant preterm birth, fetal growth restriction and perinatal complications, often hinder the timely collection and storage of autologous cord blood.^12 13^ These limitations highlight the potential for the use of allogeneic UCBC therapy,^8^ which involves donor-sourced cells and relies on Therapeutic Goods Administration (TGA)-licensed public cord blood banks. Allogeneic UCBC therapy offers advantages such as availability, standardised dosing and the potential for repeat dosing.

While research on allogeneic UCBC therapy in neonates is limited, our recent systematic review reveals that most available studies focus on umbilical cord tissue-derived mesenchymal stromal cells, particularly in the context of bronchopulmonary dysplasia,^8^ and data on other types of UCBCs, such as haematopoietic stem cells (HSCs), are limited.^8^ UCBCs which include HSCs and other progenitor cells hold immense potential for treating brain injuries,^4 5 14^ yet their application faces unique challenges due to their potential immune risk and the potential need for human leucocyte antigen (HLA) matching in this cohort of patients.^14^

To address this critical knowledge gap, we propose a study to assess the safety and feasibility of using unrelated, allogeneic, partially HLA-matched (4/6 or 5/6), UCB-derived cells in preterm infants with severe IVH and WMI. This research aims to establish a foundation for future investigations into the efficacy of allogeneic UCBCs as a novel neuroreparative therapy. Demonstrating the safety and feasibility of these therapies is an essential first step towards unlocking their potential for broader application in neonatal neurotherapeutics, potentially opening new avenues for treating brain injuries in preterm infants.

## Methods

### Design

**Figure 1:**
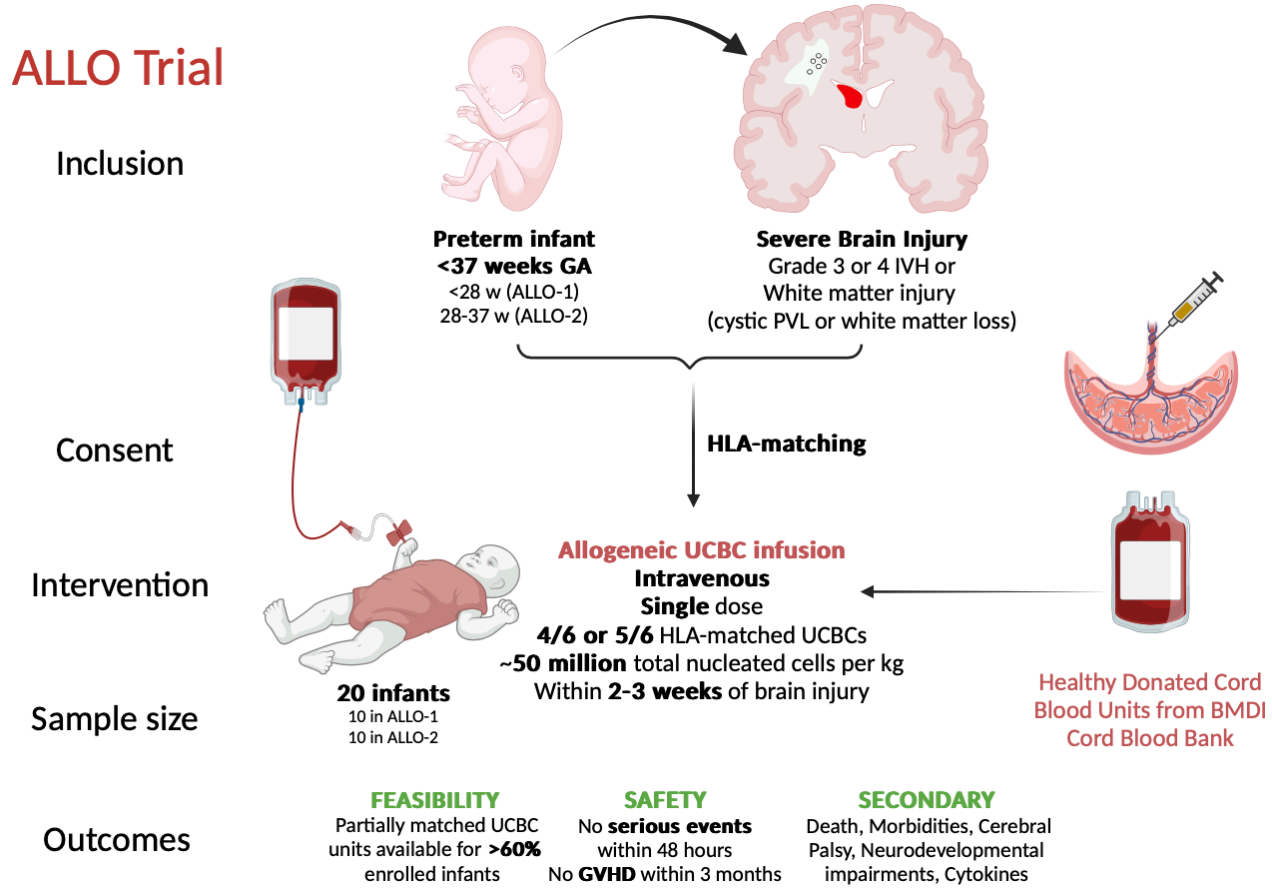
Infographic of ALLO trial. BMDI, Bone Marrow Donor Institute; GA, gestational age; GVHD, graft-versus-host disease; HLA, human leucocyte antigen; IVH, intraventricular haemorrhage; PVL, periventricular leukomalacia; UCBC, umbilical cord blood derived cell.

The ALLO trial is a phase I, safety and feasibility trial across two preterm cohorts (ALLO-1 and ALLO-2), using an open-label design (figure 1).

### Setting

The studies will be conducted at Monash Newborn, Monash Children’s Hospital, Australia in partnership with Bone Marrow Donor Institute (BMDI) Cord Blood Bank and Hudson Institute of Medical Research.

### Participants

#### Inclusion criteria

1. Preterm infants born before 28 completed weeks of gestation (up to 27+6 weeks, ALLO-1 trial) or born between 28 and 36+6 weeks of gestation (ALLO-2 trial).
2. Infants with severe brain injury detected on neonatal neuroimaging at any time after birth until 40 weeks of postmenstrual age. Severe brain injury will be considered as grade 3 (IVH with ventricular dilatation) or 4 (haemorrhagic parenchymal infarct) IVH^15^ or diffuse WMI like cystic (grade 3 or higher) periventricular leukomalacia^16^ or significant white matter loss.

#### Exclusion criteria

1. Infants with known major congenital anomalies.
2. Infants whose care is being redirected to comfort care.

Cell therapy will only be administered when preterm infants are clinically stable, as deemed by the treating physician. Also, infants should not be receiving antimicrobial therapy for confirmed or presumed neonatal sepsis, which is active at the time of cell infusion, as their course may be unpredictable and may confound adverse events falsely attributing to the cell therapy.

### Intervention details

1. HLA-matched UCBCs: unrelated, allogeneic UCBCs will be obtained from BMDI Cord Blood Bank with a HLA matching of 4/6 or 5/6 antigens (HLA-A, B and DRB1) at generic level. Given the time required for HLA matching and UCBC processing, neuroimaging (either ultrasound or MRI) will be conducted again, following recruitment, to reconfirm eligibility immediately prior to administering UCBCs.
2. Age of administration: allogeneic UCBCs will be administered via an intravenous infusion anytime from birth until 3 months of life, generally within 2–3 weeks of diagnosis of confirmed severe brain injury.
3. Cell infusion: a single allogeneic UCBC infusion will be administered intravenously over an hour.
4. UCBC dose: the target dose for the infusion is 50 million total nucleated cells (TNCs) per kg of the recipient’s body weight. This therapeutic dose is based on previous preclinical^17^ and clinical studies^18 19^ of UCBC for treatment of different types of perinatal brain injury.
5. Route: the UCBCs will be administered intravenously via a peripheral intravenous cannula.
6. UCBCs preparation and administration: the UCBCs for infusion will be obtained from BMDI Cord Blood Bank a day before transfusion, meeting Australian Network of Cord Blood Banks (AusCord) Quality and Release Criteria. The product will be released with notification to the TGA since this use of UCB is outside the scope of therapeutic indications for which the Cord Blood Bank is licensed for and temporarily stored at Monash Health Translation Precinct’s Cell Therapy and Regenerative Medicine Platform, where thawing and washing will occur on the day of administration. Cell viability (>70% viable CD34+cells) will be confirmed before administration.^12^ A sample of the patient’s serum will be collected for later cytokine analysis. Then, UCBCs suspended in dextran-albumin solution will be administered intravenously within 1 hour of thaw at a volume of 10 mL/kg over 1 hour using an infusion pump. The infusion will take place in the neonatal unit or clinical trials facility of Monash Children’s Hospital. Proper cell product custody transfer procedures will be followed throughout the process.
7. Cord blood unit selection: the selection of cord blood units is based on multiple criteria to ensure safety and suitability for use in eligible infants. Key considerations include the absence of infections (confirmed through donor screening and microbiological testing), including HIV, hepatitis B and C, human T-cell lymphotropic virus, cytomegalovirus, and other relevant viruses such as Zika, where applicable. An adequate TNC (more than 50 million cells per kg) and CD34+cell count (>70% viability) is ensured to provide a sufficient therapeutic dose, and an appropriate degree of HLA match is considered (4/6 or 5/6). Additionally, for female recipients who are Rh-negative, Rh compatibility is taken into account to prevent sensitisation. The donated cord blood units come from term infants, which naturally have high cell counts, and are generally adequate for preterm babies where the dose of 50 million TNCs per kg is targeted. While this ensures that cell dose is not a limiting factor, it remains an important selection criterion. Cord blood units with excessively high cell counts are avoided as these are typically reserved for transplantation purposes.

### Comparison

There will be no control group as this is a single-arm, open-label, phase I study. However, an exploratory analysis will compare the secondary outcomes with those from a contemporaneous cohort of preterm infants with severe brain injury born with similar inclusion/exclusion criteria during the study period admitted at Monash Health who did not receive allogeneic UCBCs.

### Outcomes

1. Primary outcomes
  1. Feasibility: allogeneic UCBC therapy will be considered feasible if there is an availability of 4/6 or 5/6 HLA-matched allogeneic UCBCs sourced from the BMDI Cord Blood Bank for >60% of eligible infants.
  2. Safety: allogeneic UCBC treatment will be considered safe if no serious adverse events occur during and within 48 hours post infusion, and if graft-versus-host disease (GVHD) is absent within the first 3 months. Safety outcomes will be reported for eligible infants receiving UCBCs, where feasibility is demonstrated. GVHD will be monitored through physical assessments at baseline, 1 month, 2 months, 3 months, 6 months, 9 months, 12 months, 18 months and 24 months. Blood tests and liver function tests will be performed before the infusion and at 24 hours, 1 week, 1 month and 3 months post infusion. Chimerism will be assessed at 1 day, 4 days, 7 days, 1 month and again at 3 months if donor cells are detected at 1 month.
2. Secondary outcomes
  1. Clinical outcomes: the following clinical outcomes will be reported for the infants who received infusion.
    - Death, until 24 months of corrected age.
    - Moderate to severe bronchopulmonary dysplasia, assessed as oxygen or positive pressure requirement at 36 weeks of postmenstrual age.
    - Severe retinopathy of prematurity, assessed as any retinopathy requiring treatment before discharge.
    - Necrotising enterocolitis, assessed as modified Bell stage 2 or more before discharge.
    - Culture-proven sepsis, bacterial or fungal, requiring antibiotic treatment for seven or more days before discharge.
    - Developmental delay, assessed based on early (General Movements, Hammersmith Neonatal/Infant Neurological Examination at 3–4 months corrected age)^20 21^ or late (Bayley Scales of Infant and Toddler Development, fourth edition (Bayley-4) at 24–36 months corrected age) neurodevelopmental assessments.
    - Cerebral palsy, assessed based on early (General Movements, Hammersmith Neonatal/Infant Neurological Examination at 3–4 months corrected age) or late (Gross Motor Function Measure-66 at 12 months corrected age, Bayley-4 and Gross Motor Function Classification System Expanded and Revised at 24–36 months corrected age) neurodevelopmental assessments.
    - Blindness, assessed any time before 18–24 months corrected age.
    - Deafness, assessed any time before 18–24 months corrected age.
  2. Laboratory outcomes: a broad array of both inflammatory and anti-inflammatory cytokines will be assessed on the infant’s serum, including (but not limited to) interleukin (IL)−6, IL-1β, IL-10, tumour necrosis factor-alpha and monocyte chemoattractant protein-1, just before UCBC administration and 1 day post administration.

### Recruitment and identification of potential participants

The study team will conduct weekly reviews of head ultrasound or MRI scans for preterm infants in the neonatal intensive care unit at Monash Children’s Hospital to identify cases of severe preterm brain injury. On detection of severe brain injury and confirmation of study eligibility, the infant’s family will be approached for consent.

### Consent

The study team will provide verbal and written information about the trial to the parent/guardian. Sufficient time will be allowed for consideration before obtaining voluntary written consent (online supplemental information). The consent process will be documented and a copy provided to the family. Participation, non-participation and ineligibility will be recorded in the enrolment log and electronic medical record.

### Sample size

This pilot study is designed to assess the feasibility and safety of the intervention, with the goal of advancing to a larger, more definitive efficacy trial if successful. We defined 60% feasibility in HLA-matching as an acceptable threshold, while 30% feasibility was considered unacceptable.^22^ Based on 80% power and a significance level of 0.05 and a statistical significance threshold of 47.8%, the required sample size was determined to be 18 patients. To account for a potential 10% loss to follow-up, we will include 20 patients in the study, ensuring the trial is well-positioned to provide meaningful insights for future research. We plan to enrol 10 patients in each of two strata: ALLO-1 trial (<28 weeks gestation) and ALLO-2 trial (28 to 36+6 weeks gestation). Patients will receive UCBCs at the specified dose range if appropriate HLA matching is achieved. It is important to note that the number of preterm infants receiving the cell infusion may be less than 20, as some eligible infants may not meet HLA matching criteria or may not survive to receive the infusion.

### Study timeline

The ALLO trial commenced recruitment in May 2024 and will continue until the target enrolment of 20 participants (10 in ALLO-1 and 10 in ALLO-2) is fulfilled. Follow-up assessments will extend to 24–36 months corrected age for enrolled participants to evaluate long-term outcomes.

### Statistical analysis

Descriptive statistics will be used to report demographic data and outcomes. The categorical variables will be described as frequency and percentage with their 95% CIs and continuous variables as median (IQR) and mean (SD), depending on the normality of the data, which will be determined by the Shapiro-Wilk test. The data will be analysed using STATA V.18.0 (StataCorp, College Station, Texas, USA). Paired t-tests or Wilcoxon signed-rank tests will be performed to compare the laboratory parameters before and after infusion.

### Safety monitoring and reporting

Events will be categorised according to the NHMRC 2016 Safety, Monitoring and Reporting recommendations^23^ and reported accordingly. All serious events, including those resulting in death, life-threatening conditions, hospitalisation or prolonged hospitalisation or persistent or significant disability or incapacity, will be promptly reported to the Monash Human Research Ethics Committee (HREC) and the data safety monitoring board (DSMB). The DSMB will include a neonatologist and a cell therapist or haematologist/oncologist. In the event of a serious adverse event, both the DSMB and HREC will be consulted to assess whether it is safe to continue the trial.

An interim safety and feasibility analysis is planned after the enrolment of five patients in each group. If necessary, the DSMB and HREC will conduct an independent review of the trial’s conduct, progress and any serious events related to cell therapy to determine if the trial should be prematurely terminated.

### Patient and public involvement

Input of people with lived experience of preterm neonatal care and/or cerebral palsy has been incorporated into the trial design, including the selection of outcomes, the language used in parent information sheets and consent forms and plans for data dissemination and sharing. There is no industry involvement in this trial. At Monash Health (trial sponsor), relevant supporting departments have been engaged to assess feasibility, resource requirements, potential time constraints and associated costs.

## Discussion

The ALLO trial is a groundbreaking initiative aimed at assessing the feasibility and safety of allogeneic UCBC therapy in preterm infants with severe brain injury—a leading cause of long-term neurodevelopmental impairment. The majority of affected surviving infants go on to develop cerebral palsy, and effective treatment options remain scarce.^2^ This phase I study addresses a critical unmet need by investigating allogeneic UCBCs as a potential neuroreparative therapy, building on preclinical evidence of their anti-inflammatory and neuroprotective effects.

Allogeneic UCBC therapy carries theoretical risks of immune-related adverse events, such as GVHD, inherent to any allogeneic cell therapy due to donor-recipient immunological differences. However, UCBCs have a safer immunological profile compared with other allogeneic cell types, attributed to their low immunogenicity and the immature immune system of preterm infants. The ALLO trial mitigates these risks by using a single infusion of partially HLA-matched (4/6 or 5/6) UCBCs, which significantly reduces the already minimal risk of GVHD. The study targets a high-risk cohort with severe brain injury, where the natural course often leads to death or significant disability. Our prior work highlights that many infants with severe IVH or WMI are redirected to comfort care due to poor prognosis,^2^ underscoring the limited therapeutic options. Given this context, the potential benefits of allogeneic UCBC therapy may balance or even outweigh the theoretical risks, offering hope for improved outcomes in this vulnerable population.

The ALLO trial’s robust study design ensures meticulous risk assessment and management. Conducted at Monash Children’s Hospital with close collaboration among neonatologists, haematologists and cell therapy experts, the trial includes intensive follow-up to detect and address potential adverse events early. The UCBC infusion, administered intravenously at 10 mL/kg over 1 hour in the neonatal intensive care unit, mirrors protocols from our prior autologous CORD-SaFe study,^13^ ensuring safety through continuous monitoring of vital signs and clinical status. A paediatric haemato-oncologist oversees GVHD evaluation, which, while unlikely due to the neonatal immune profile and single-dose approach, is rigorously monitored through physical assessments and blood tests at multiple time points. This approach will likely help us identify any cases of GVHD early, enabling prompt and effective treatment with immunosuppressive agents, such as corticosteroids, tailored to minimise risks like infection susceptibility in neonates.

While the primary focus of the ALLO trial is feasibility and safety, it holds promise for demonstrating efficacy in mitigating the devastating effects of severe preterm brain injury. The 2–3 week intervention window post diagnosis balances the need for early administration to maximise neuroprotective and anti-inflammatory benefits, as supported by preclinical studies, which showed that UCBC administration within days to weeks post injury prevented white matter damage in animal models.^17^ This timeframe also accommodates logistical requirements, such as HLA matching and cell preparation, while ensuring infants achieve clinical stability. By establishing that allogeneic UCBCs can be sourced for >60% of eligible infants and administered without serious adverse events or GVHD, the trial paves the way for larger efficacy studies. In summary, the ALLO trial addresses a critical unmet need in neonatal care by rigorously evaluating the feasibility and safety of allogeneic UCBC therapy, with the potential to transform outcomes for preterm infants with severe brain injury.

## Ethics and dissemination

### Ethics

This study will be conducted in accordance with the approved protocol (V.2.2, 2 December 2024) (online supplemental information) and any amendments, the conditions set by Monash HREC and the NHMRC National Statement on Ethical Conduct in Human Research (2007, updated May 2018).^24^ A full ethics approval has been obtained (RES-23-0000-297A), including approval for the research use of allogeneic cord blood from compassionate donations provided by healthy donors, facilitated through the BMDI Cord Blood Bank, which operates as part of the AusCord network. The committee has endorsed the inclusion of patients with severe brain injury after carefully weighing the potential risks and benefits.

### Dissemination

The study design and findings will be disseminated via scientific conferences, publication in peer-reviewed journals and social media platforms. No identifiable participant data will be reported. Participant families will each receive a summary letter of study outcomes or the publication at the conclusion of the study if desired.

## Supplementary material

10.1136/bmjopen-2025-100389 online supplemental file 1
