# Supplementary material for: Safety and feasibility of allogeneic cord blood-derived cell therapy in preterm infants with severe brain injury (ALLO trial): a phase-1 trial protocol
Source: BMJ Open. 2025 Jun 18;15(6):e100389. doi: 10.1136/bmjopen-2025-100389 (PMC12182109; doi:10.1136/bmjopen-2025-100389)
Supplement: online supplemental file 1 [file bmjopen-15-6-s001.docx]

**SUPPLEMENTAL INFORMATION**

| **Table of Contents** | **Page number** |
| --- | --- |
| Protocol | 2-32 |
| Participant information consent form | 33-45 |
| Case reporting form | 46-53 |

**Research Protocol**

**Safety and Feasibility of Allogeneic Cord Blood-Derived Cell Therapy in Preterm Infants with Severe Brain Injury (ALLO Trial)**

**Short title:** Allogeneic cord blood cell therapy for preterm brain injury

**Type of trial**: Phase-I, single-arm, feasibility and safety trial

**Protocol Version:** 2.2

**Trial Sponsor:** Monash Health

**Coordinating Principal Investigator**: Associate Professor Atul Malhotra

**PhD Student/ Principal Investigator:** Dr Abdul Razak

**Associate Investigators**: Dr Michelle Martin, Dr Lindsay Zhou, Dr Madison Paton, Associate Professor Ngaire Elwood, Dr Courtney McDonald, Professor Suzanne Miller, Professor Rod Hunt

**Clinical Location**: Monash Medical Centre & Monash Children's Hospital, Monash Health, Clayton, VIC

**Cord Blood Bank & Cell Release Facility**: BMDI Cord Blood Bank, MCRI, Parkville, VIC

**Research Laboratory**: The Ritchie Centre, Monash University & Cell Therapies and Regenerative Medicine Platform, Hudson Institute of Medical Research, Clayton, VIC

**Trial statement**: The study will be conducted according to this protocol and will conform to good clinical practice and all applicable regulatory requirements.

**PROTOCOL SYNOPSIS**

| **Title** | **Safety and Feasibility of Allogeneic Cord Blood-Derived Cell Therapy in Preterm Infants with Severe Brain Injury** |
| --- | --- |
| **Objectives** | Primary   1. To evaluate feasibility of availability of allogeneic umbilical cord blood cells (UCBCs) in surviving preterm infants with severe brain injury. 2. To evaluate safety of administration of allogeneic UCBCs in surviving preterm infants with severe brain injury.   Secondary   - 1. To evaluate the effect of administration of allogeneic UCBCs on short and long-term clinical outcomes.   2. To evaluate the effect of administration of allogeneic UCBCs on immune responses, as measured by cytokine levels. |
| **Outcomes and outcome measures** | Primary outcomes   - 1. Feasibility: Availability of at least 4/6 HLA-matched allogeneic UCBCs sourced from the BMDI Cord Blood Bank for >60% of eligible infants.   2. Safety: Absence of serious adverse events related to UCBCs administration during and for 48 hrs post-infusion and the absence of graft versus host disease (GVHD) in the first three months after cell infusion.   Secondary outcomes   - 1. Clinical outcomes      1. Death, until 24 months of corrected age      2. Moderate to severe bronchopulmonary dysplasia, assessed at 36 weeks of postmenstrual age      3. Severe retinopathy of prematurity, assessed before discharge      4. Necrotising enterocolitis, assessed before discharge      5. Culture proven sepsis, assessed before discharge      6. Developmental delay, assessed anytime until 24 months of corrected age      7. Cerebral palsy, assessed anytime until 24 months of corrected age      8. Blindness, assessed any time until 24 months corrected age      9. Deafness, assessed any time until 24 months corrected age   2. Laboratory outcomes: Effect of allogeneic UCBCs on infant’s cytokines levels, including interleukin 1b (IL-1b), IL-6, Tumour Necrosis Factor (TNF-α), and IL-10 just before UCBC administration and 1-day and 7-day post administration |
| **Trial population** | 20 preterm infants with severe brain injury, with 10 each in each stratum (<28 weeks, ALLO-1 trial and 28-37 weeks, ALLO-2 trial), will be enrolled to receive UCBCs, if appropriate matching is achieved |
| **Description of sites enrolling participants** | This trial will only recruit participants from Monash Children’s Hospital |
| **Description of interventions** | One intravenous infusion of 4/6 or higher HLA-matched allogeneic UCBCs obtained from BMDI Cord Blood Bank at a dose of 50 million cells per kg |
| **Trial duration** | 3-5 years |
| **Participant duration** | 24 months post-intervention |

**GLOSSARY OF ABBREVIATIONS**

| **ABBREVIATION** | **TERM** |
| --- | --- |
| AE | Adverse event |
| AR | Adverse reaction |
| BSID | Bayley Scales of Infant Development |
| CA | Corrected age |
| CBU | Cord blood unit |
| DSMB | Data safety monitoring board |
| FBE | Full blood examination |
| GVHD | Graft versus host disease |
| HLA | Human leucocyte antigen |
| HREC | Human Research Ethics Committee |
| IVH | Intraventricular haemorrhage |
| LFT | Liver function tests |
| MRI | Magnetic resonance imaging |
| PVL | Periventricular leukomalacia |
| SAE | Serious adverse event |
| SAR | Serious adverse reaction |
| TEA | Term-equivalent age |
| TNCs | Total nucleated cells |
| UCBC | Umbilical cord blood cells |

**INVESTIGATOR AGREEMENT**

I have read the protocol entitled ‘**Safety and Feasibility of Allogeneic Cord Blood-Derived Cell Therapy in Preterm Infants with Severe Brain Injury**.’

By signing this protocol, I agree to conduct the clinical trial, after approval by a Human Research Ethics Committee or Institutional Review Board (as appropriate), in accordance with the protocol, the principles of the Declaration of Helsinki and the good clinical practice guidelines adopted by the TGA [Integrated Addendum to ICH E6 (R1): Guideline for Good Clinical Practice E6 (R2), dated 9 November 2016 annotated with TGA comments].

Changes to the protocol will only be implemented after written approval is received from the Human Research Ethics Committee or Institutional Review Board (as appropriate), except for medical emergencies.

I will ensure that trial staff fully understand and follow the protocol and that evidence of their training is documented on the trial training log.

| **Name** | **Role** | **Signature and date** |
| --- | --- | --- |

1. **ADMINISTRATIVE INFORMATION**
   1. ***Trial registration:*** This trial will be registered on the ClinicalTrials.gov and Australian New Zealand clinical trial registry, and the registration number provided at that time.
   2. ***Trial sponsor***

| **Trial sponsor** | Monash Health |
| --- | --- |
| **Contact name** | Associate Professor Atul Malhotra |
| **Address** | 246 Clayton Road, Clayton, VIC, 3168 |
| **Sponsor-Investigator** | Associate Professor Atul Malhotra |

- 1. ***Expected duration of the study:*** Approximately every year, 6-8 preterm infants admitted to the neonatal intensive care unit at Monash Children’s Hospital are anticipated to have a severe brain injury; therefore, the recruitment is expected to span over 2.5 years. Each participant will be followed up for 3 months after the cell infusion to determine the safety outcome. Data analysis and interpretation are expected to take a further 3 months. Therefore, the study is predicted to take a total of 3 years to determine the safety and feasibility of the treatment. To determine long-term effects related to the treatment, each participant will be further followed up to 24 months post-infusion.
  2. ***Roles and contribution***

| Name | Summary of contribution |
| --- | --- |
| *Associate Professor Atul Malhotra*,  Neonatologist, Head, Early Neurodevelopment Clinic, Monash Children’s Hospital;  Associate Professor (Research), Monash University | Co-ordinating principal investigator  Scientific design and planning  Patient recruitment and follow-up  Interpretation and analysis |
| *Dr Abdul Razak*  Doctoral student, Monash University  Neonatal fellow, Monash Children’s Hospital | PhD student, principal investigator  Scientific design and planning  Patient recruitment and follow-up  Interpretation and analysis |
| *Dr Michelle Martin*  Haemato-oncologist  Monash Children’s Hospital | Scientific design and planning  Patient recruitment and follow-up  Transplant physician responsible for sign off on infusion  Interpretation and analysis |
| *Dr Lindsay Zhou*  Neonatologist, Monash Children’s Hospital; Doctoral student, Monash University | Scientific design and planning  Patient recruitment and follow-up  Interpretation and analysis |
| *Dr Madison Paton,* Scientist  Research Fellow, Cerebral Palsy Alliance Research Institute; The University of Sydney | Scientific design and planning Interpretation and analysis |
| *Associate Professor Ngaire Elwood*  Scientist, Blood Development, Murdoch Children’s Research Institute;  Director, BMDI Cord Blood Bank | Scientific design and planning Interpretation and analysis  Director of BMDI Cord Blood Bank – responsible for final sign off for release of selected cord blood unit, in accordance with release criteria.  Notification to TGA of exceptional release of cord blood unit. |
| *Dr Courtney McDonald*  NHMRC Peter Doherty/Cerebral Palsy Alliance Early Career Research Fellow  The Ritchie Centre  Hudson Institute of Medical Research  Monash University | Scientific design and planning Interpretation and analysis |
| *Professor Suzanne Miller*  Head, The Ritchie Centre  Department of Obstetrics and Gynaecology, Monash University  Hudson Institute of Medical Research | Scientific design and planning Interpretation and analysis |
| *Professor Rod Hunt*  The Financial Markets Foundation for Children Chair, Neonatal Paediatrics  Paediatrics Education & Research, Monash University  Director of Research, Victoria, Cerebral Palsy Alliance  Neonatologist, Monash Children's Hospital | Scientific design and planning Interpretation and analysis |

- 1. ***Stakeholder involvement***

*Consumer advisory group involvement (Monash newborn research)*: We have obtained consumer feedback on the trial design, outcomes, language on the parent information sheet and consent forms, dissemination and data sharing plans.

*Industry involvement*: There is no industry involvement in this trial.

*Public infrastructure involvement*: Australia has a public cord blood banking network called AusCord, funded via the Commonwealth Health Committee, with service contracts via the Australian Bone Marrow Donor Registry. The BMDI Cord Blood Bank operates as a consortium between the Murdoch Children’s Hospital, the Royal Children’s Hospital and Fight Cancer Foundation, and is one of three public cord blood banks in Australia that together form the AusCord Network. The bank relies on voluntary, compassionate donations of cord blood from healthy donors to be used for the treatment of unrelated patients around the world. The cord blood units (CBU) for treatment in this trial will be obtained from the BMDI Cord Blood Bank. AusCord now has the capacity to release CBU to Therapeutic Good Administration-notified clinical trials. The study team has engaged with BMDI Cord Blood Bank in the development of the trial with a particular focus on aligning study outcomes with AusCord’s requirements for an evidence base.

*Internal site involvement*: At Monash Health, supporting departments have been engaged to determine feasibility, possible time burdens/limitations and costs.

1. **INTRODUCTION**
   1. ***Trial rationale and aim***

Severe intraventricular haemorrhage (IVH) and diffuse white matter injury, like cystic periventricular leukomalacia (PVL) are significant neonatal neurological morbidities in preterm infants. Recent data from the Australian and New Zealand Neonatal Network shows that the risk of severe IVH is trending down over the last few years; however, it is still substantial, with 5 percent of babies less than 32 weeks’ gestation and 11.6 percent less than 27 weeks’ gestation suffering from severe IVH.^1^ Infants with severe IVH and cystic PVL are at risk of adverse neurodevelopmental outcomes.^2^ Approximately 17.5 percent and 30 percent of survivors with severe IVH have a developmental delay and cerebral palsy, respectively. Umbilical cord blood cell (UCBC) therapy is one promising early treatment option for surviving preterm infants with this injury. It may mitigate the risk of cerebral palsy or neurodevelopmental impairments in these infants. Autologous UCBC therapy may be feasible and an option in some infants, but it may be challenging in a few infants,^3^ and hence allogeneic UCBC therapy is an attractive option. However, the safety and feasibility of allogeneic UCBC administration in preterm infants are yet to be determined. Therefore, in this study, the aim is to investigate the feasibility and safety of single intravenous infusion of at least 4/6 or higher human leucocyte antigen (HLA) matched allogeneic UCBCs to surviving preterm infants with severe IVH and cystic PVL.

- 1. ***Background***

*Overview*: UCBC therapy has been investigated in clinical research for several neonatal morbidities;^4^ however, much work is still required. While autologous trials of UCBC therapy targeting neurological conditions, such as hypoxic-ischemic encephalopathy and preterm brain injury, are being investigated, research on allogeneic UCBC therapy for preterm brain injury is still preliminary. Research on allogeneic UCBC therapy is more crucial as preliminary data from the ongoing CORD-SAFE (autologous UCBC therapy in extremely preterm infants) feasibility study demonstrates that while autologous UCBC therapy may be an option for some babies, many will require a donor-sourced cell therapy as preterm birth often makes the collection of autologous UCBCs challenging.^3^ Notably, collecting UCBCs in preterm infants who deliver quickly or infants born in the context of antepartum haemorrhage and chorioamnionitis is challenging. In addition, these infants are at greater risk of severe IVH as they are likely born in poor condition, and UCBCs may not have been collected in some of these situations.

*Pros and cons of allogeneic UCB therapy*: Allogeneic UCBC therapy offers many advantages. It is readily available, can be targeted to efficacious doses, and enables repeat dosing. However, there are a few disadvantages, primarily the risk of a host-mounted immune response, which may negate the benefits due to clearance of transfused UCBCs or may carry unknown risks, which sometimes can be severe. It is unclear whether this risk in young preterm infants is similar to children and adults, as research in this group is largely lacking. In addition, HLA-matching is an important process for allogeneic cell therapy, but getting a full 6/6 match may not be feasible. On the other hand, a slight mismatch is probably desirable, as non-homologous use of allogeneic cells is not intended to engraft in the long term.

*Evidence on allogeneic UCBC therapy*: So far, one study has examined and demonstrated the safety and feasibility of a single dose of intraventricular allogeneic cord mesenchymal stem cells in nine preterm infants with severe IVH.^5^ Similarly, in another case report, one preterm infant with severe IVH was administered an intraventricular and intravenous dose of allogeneic cord mesenchymal stem cells,^6^ resulting in a normal neurological outcome. Overall, research on allogeneic UCBC therapy for targeting preterm brain injury is limited and urgently required, as highlighted in our recent systematic review of allogeneic cell therapy in neonates (ongoing).

*Mechanisms of UCBCs in preterm brain injury*: UCBC therapy delivered intravenously for preterm brain injury is postulated to work via anti-inflammatory and anti-apoptotic properties, with effects on cellular survival and angiogenesis.^7,8^ It also protects the blood-brain barrier and vulnerable oligodendrocyte populations.^8^ A recent systematic review has shown that UCBC therapy in preclinical brain injury models significantly improved outcomes across many domains, including neuroinflammation, apoptosis, microglial activation, astrogliosis, motor function, and neuronal and oligodendrocyte number.^9^ Furthermore, as highlighted in another systematic review, these benefits may have greater efficacy in the preclinical injury model of IVH than hypoxia-ischemia and may also have differential efficacy based on the cell type and route.^10^ Finally, these beneficial effects are also seen in the clinical context, where UCBCs administration have shown to be efficacious in improving motor outcomes in children with cerebral palsy.^11^ In this study, similar gains are anticipated with allogeneic UCBCs administration in preterm infants with severe brain injury with an optimised treatment applied much earlier, closer to the timing of the injury.

- 1. ***Risk-benefit analysis***

The proposed use of allogeneic cell therapy in preterm infants with severe IVH and cystic PVL carries potential risks and benefits. Steering a proper risk-benefit analysis is challenging as the exact incidence of these risks and benefits remains unknown, and hence this feasibility and safety study is being conducted.

*Risk with allogeneic cell therapies*: Graft versus host disease (GVHD) is a potential risk to consider in all allogeneic cell therapy applications. The exact occurrence and severity of GVHD in preterm infants are currently unknown. However, it is anticipated that the risk of GVHD will be minimal or negligible when utilizing cord blood-derived therapy applications for injury mitigation in neonatal neurology trials.

*Dissimilar contextual setting*: It is crucial to acknowledge that the purpose of administering umbilical cord blood cells is to introduce them into the system with the aim of exerting their therapeutic effects on the targeted disease via their paracrine effects utilization, which is distinct from their use in haematology trials for transplant procedures. In the latter, the therapies are aimed for cells to engraft, survive, and proliferate. Several key differences exist between haematology and neonatal neurology trials in terms of the study population, intervention, and setting. Generally, neonatal trial participants possess a functioning immune system for their gestational age, and progenitor or stem cells are expected to remain in their system for a shorter duration to achieve the intended benefits.^8^ Whereas, haematology trial participants are often unwell, with haematologic malignancies and compromised immune systems. These significant differences may impact the likelihood of GVHD occurrence. GVHD risk remains a significant concern in the field of transplantation medicine; however, no similar risk has been reported in neonatal trials involving cell therapies outside of transplantation. Our systematic review on allogeneic cell therapy, encompassing all trials involving allogeneic cell therapy in the neonatal period, highlights two reports documenting GVHD occurrence in transplantation-focused cell therapy applications. Conversely, no reports of GVHD associated with cell therapy applications for non-transplantation purposes were identified. Additionally, our review identified a study, similar to the proposed study, which documented the absence of GVHD in the preterm population following cord blood-derived mononuclear cell therapy.^12^

As previously mentioned, it is not expected for cells to engraft and persist in the body in neonatal cell therapy trials.^8,13^ Preclinical studies indicate that administered cells seldom remain in the brain beyond few days to weeks.^8,13,14^ Likewise, clinical data shows that even in cases where there is a perfect 12/12 human leukocyte antigen (HLA) match, the infused cells are eliminated within approximately three months in almost all participants.^15^ Overall, the theoretical risk of GVHD from allogeneic cell therapy applications involving the use of cord blood-derived therapies in the preterm or neonatal population is highly unlikely outside of transplantation settings, as evidenced by a systematic review that found no instances of GVHD in children and adults treated with allogeneic UCB-derived total nucleated cells (TNCs) or mononuclear cell therapy.^12^

*Cord blood-derived therapies poses low risk*: The risk of graft versus host disease (GVHD) varies among allogeneic cell therapy applications, depending on the source of therapy. In comparison to peripheral blood or bone marrow cell therapies, the risk of GVHD following cord blood-derived therapies is expected to be low. This can be attributed to the distinctive characteristics of cord blood. UCBCs possess a favourable profile that aids in preventing GVHD at various stages. The immune cells derived from the cord blood are predominantly naive (CD45RA+),^16^ as their exposure to environmental pathogens and vaccines is limited compared to adults. These CD45RA+ cells display reduced immunological responsiveness towards alloantigens. In addition, the allogeneic activation of T cells is impaired.^17,18^ Likewise, the antigen-presenting activity of dendritic cells derived from cord blood is immunologically defective.^19^ As well, cord blood T cells' Th1 immune responses are diminished.^20^ Furthermore, the cytotoxic effect of CD8+ T cells is lowered.^21,22^ Despite UCBCs having several deficient processes or impaired responses, they exhibit heightened immune tolerance towards HLA mismatch,^23^ which can be attributed to the presence of regulatory T cells^24^ and mesenchymal stem cells.^25^ These cells effectively modulate immune responses and thereby suppress harmful reactions. Additionally, our study adopts a cautious approach by utilising one infusion, aligning with literature supporting a lower risk of GVHD with reduced/single exposure. Lastly, HLA-matching plays a crucial role in reducing the risk of GVHD, and a 4/6 match is presumed to reduce the risk in a substantial manner.

*Other risks involved*: Acknowledging the other potential risks associated with the therapy, such as infusion-related reactions, including anaphylaxis, local site reactions, infection, and unforeseen reactions, is important. It is also crucial to emphasize that these risks are rare and established protocols and safety measures are in place to mitigate them. Furthermore, with diligent monitoring and timely interventions, these risks can be effectively managed and mitigated.

*Benefits of therapy*: On the other hand, the potential benefits of allogeneic therapy hold a promising future. Although the specific impact of allogeneic cell therapy in preterm infants with severe brain injury is currently unknown, the evidence from preclinical and clinical cord blood derived cell therapy research suggests a promising avenue for exploration and improvement. One of the mentioned studies using allogeneic cell therapy in neonates showed favourable changes in the natural course of the neurological disorder and improved neurological outcomes.^26^ The possibility of reducing neurodevelopmental impairments and cerebral palsy in preterm infants with severe brain injury who are at substantial risk holds immense clinical and economic significance. Our local data from 2014 to mid-2022 indicate that a significant proportion of babies with severe IVH (one of the indications of this study) experience either mortality (43 percent), possibly from sickness or redirection of care, or adverse neurodevelopmental outcomes (pending analysis). Treating neurological disability or cerebral palsy can significantly burden healthcare systems and families financially and emotionally. Allogeneic cell therapy has the potential to revolutionize the approach to this condition, reducing the incidence of long-term morbidities and alleviating the associated burdens.

*Balancing the risk vs. benefit*: In summary, it is important to recognize the potential risk of GVHD, which is highly unlikely, as well as other adverse effects associated with allogeneic cord blood cell therapy. However, it is crucial to emphasize the significant potential benefits of this innovative approach. The opportunity to potentially reduce the occurrence or severity of cerebral palsy, neurodevelopmental disabilities, and sensory impairments warrants careful consideration. While the ultimate goal is to establish the effectiveness of this treatment, it is imperative to prioritize the initial stages of assessing its feasibility and safety. Given the potential risks involved, it may be judicious to focus the study on a cohort of preterm infants who face the highest risk of future neurological disabilities. This approach allows for a balanced consideration of both the inherent risks and potential benefits.

1. **TRIAL OBJECTIVES**
   1. ***Primary*:** To evaluate the safety and feasibility of administration of single-dose, at least 4/6 or higher HLA-matched allogeneic UCBC therapy in preterm infants with severe brain injury.
   2. ***Secondary:***
      1. To evaluate the effect of administration of allogeneic UCBCs on short and long-term clinical outcomes of enrolled preterm infants.
      2. To evaluate the effect of administration of allogeneic UCBCs on immune responses as measured by cytokine levels in enrolled preterm infants.
2. **METHODS**
   1. ***Design***

This is an open-label, phase-I, single-arm, feasibility, and safety study.

- 1. ***Setting***

The studies will be conducted at Monash Newborn, Monash Children’s Hospital, and Monash Medical Centre, Monash Health, in partnership with BMDI Cord Blood Bank, Parkville and Hudson Institute of Medical Research, Clayton.

- 1. ***Ethics***

The study will be conducted in compliance with the approved protocol/amendment(s), conditions of Monash Health Human Research Ethics Committee **(**HREC) approval, and the NHMRC National Statement on Ethical Conduct in Human Research 2007.^27^ A letter of study approval will be obtained from HREC prior to the commencement of the trial. Further, any amendments to the study will be submitted to HREC for approval prior to being implemented.

- 1. ***Participants***
     1. Inclusion criteria
        1. Preterm infants born before 28 completed weeks of gestation (up to 27+6 weeks, ALLO-1 trial) OR born between 28 and 36+6 weeks of gestation (ALLO-2 trial) AND
        2. Severe brain injury detected on neonatal neuroimaging any time after birth. Severe brain injury will be considered as grade 3 (IVH with ventricular distension) or 4 (parenchymal haemorrhagic infarct) IVH,^28^ and diffuse white matter injury like cystic (grade 3) PVL^29^ or significant white matter loss. If there is uncertainty regarding the severity of preterm brain injury, a discussion will be conducted with the clinical and radiological teams to assess and confirm whether the injury qualifies as severe.
     2. Exclusion criteria
        1. Infants with known major congenital anomalies OR
        2. Infants whose care is being redirected to comfort care.

Cell therapy will only be administered when preterm infants are clinically stable, which is deemed by the treating physician. Also, infants should not be receiving antimicrobial therapy for confirmed or presumed late-onset neonatal sepsis, which is active at the time of cell infusion, as their course may be unpredictable and may confound adverse events falsely attributing to the cell therapy.

- 1. ***Recruitment and identification of potential participants***

Head ultrasound or magnetic resonance imaging (MRI) scans for preterm infants admitted to the neonatal intensive care unit at Monash Children’s Hospital will be reviewed by the study team daily to identify severe brain injury. If a severe brain injury is identified on the scan, the infant’s family will be approached for consent, provided all the additional criteria are met. The eligibility will be further re-confirmed before administering the intervention.

- 1. ***Consent***

The study team will explain verbally about the trial and will also provide the Participant Information and Consent Form to the parent or legal guardian. The participant's family will be given sufficient time for consideration and family discussion for providing written, voluntary consent that is free from coercion. The team member obtaining the informed consent will also sign the informed consent form. A copy of the consent form will be given to the parent or legal guardian. Documentation about the participant’s participation and voluntary consent will be noted in the electronic medical record. Participants’ families who wish not to participate in the trial will be recorded. Similarly, eligible participants who are not approached and participants who have a severe brain injury but are otherwise ineligible will be recorded in the enrollment log.

- 1. ***Sample size***

A minimum of 20 preterm infants, with 10 each in each stratum (<28 weeks, ALLO-1 trial and 28 to 36+6 weeks, ALLO-2 trial), will be enrolled to receive UCBCs at the dose range mentioned below, if appropriate HLA matching is achieved. This sample size is consistent with other cell therapy trials in newborn infants. The number of preterm infants recruited for the safety outcome (cell infusion) may be less as some eligible infants may not suffice the criteria required for HLA matching or may not survive to receive the infusion, but the aim is to assess feasibility for at least 10 infants in each stratum.

- 1. ***Intervention details***
     1. HLA matching: Allogeneic UCBCs will be obtained from BMDI Cord Blood Bank with an HLA matching of at least 4/6. As HLA matching and processing UCBCs may be time-consuming, the eligibility (neuroimaging) will be reconfirmed before administering the allogeneic UCBCs after successful HLA matching.
     2. Age of administration: Allogeneic UCBCs will be administered via IV infusion anytime from birth until three months of life, generally within 2-3 weeks of diagnosis of confirmed severe preterm brain injury.
     3. Cell infusion: Only one allogeneic UCBC infusion will be administered intravenously.
     4. UCBC dose: The infusion is aimed for 50 million total nucleated cells (TNCs) per kilogram of body weight will be administered. This therapeutic dose is based on preclinical^30^ and clinical studies^5,6^ of UCBC for different types of perinatal brain injury.
     5. Route: The UCBCs will be administered intravenously via a peripheral intravenous cannula.
  2. ***Comparison***

There will be no control group as this is a single-arm, open-label, phase-I study.

- 1. ***Outcomes***
     1. Primary outcomes
        1. Feasibility: Allogeneic UCBC therapy will be considered feasible if there is an availability of at least 4/6 HLA-matched allogeneic UCBCs sourced from the BMDI Cord Blood Bank for >60% of eligible infants.
        2. Safety: Allogeneic UCBCs will be considered as safe if there are no serious adverse events related to UCBCs during and for 48 hrs post-infusion and the absence of GVHD in the first three months after cell infusion. The safety outcomes will be reported for eligible infants where feasibility is demonstrated, and allogeneic UCBCs are administered.
     2. Secondary outcomes
        1. Clinical outcomes: The following clinical outcomes will be reported for the infants who received infusion.
           1. Death, until 24 months of corrected age
           2. Moderate to severe bronchopulmonary dysplasia, assessed as oxygen or positive pressure requirement at 36 weeks of postmenstrual age
           3. Severe retinopathy of prematurity, assessed as any retinopathy requiring anti-VEGF or laser treatment before discharge
           4. Necrotising enterocolitis, assessed as bell stage 2 or more before discharge
           5. Culture-proven sepsis, bacterial or fungal, requiring antibiotics treatment for 7 or more days before discharge
           6. Developmental delay, assessed based on early **(**General Movements, Hammersmith Neonatal/Infant Neurological Examination at 3-4 months corrected age) or late (Bayley Scales of Infant Development, (BSID) IV at 18-24 months corrected age) neurodevelopmental assessments
           7. Cerebral palsy, assessed based on early **(**General Movements, Hammersmith Neonatal/Infant Neurological Examination at 3-4 months corrected age) or late (BSID-IV and Gross Motor Function Classification System Expanded and Revised at 18-24 months corrected age) neurodevelopmental assessments
           8. Blindness, assessed any time before 18-24 months corrected age
           9. Deafness, assessed any time before 18-24 months corrected age
        2. Laboratory outcomes: A broad array of cytokines will be assessed on the infant’s serum, including interleukin 1b (IL-1b), IL-6, Tumour Necrosis Factor (TNF-α), and IL-10 just before UCBC administration and 1-day and 7-day post administration.
  2. ***UCBCs administration***

The criteria for eligibility will be confirmed on the day of infusion. The cell product will be obtained from the BMDI Cord Blood Bank a day before the transfusion. CBU released from the Cord Blood Bank for this study will have undergone all pre-release testing as routinely performed for release for bone marrow transplant, and have met compliance with the AusCord Quality & Release Criteria. The CBU will be released under “Exceptional Release” and the TGA notified as the bank is not licensed for the release of unrelated allogeneic non-homologous use. The product will be thawed at Monash Health Translation Precinct's Cell Therapy and Regenerative Medicine Platform. After thawing, washing of cells (to remove DMSO, a cryopreservant) will be undertaken using Dextran-Albumin and resuspended in 4% albumin as per standard clinical practice. Cell viability (>70% viable CD34+ cells, in accordance with the AusCord Quality & Release Criteria.) will be determined before administration. UCBCs will be administered intravenously (through a peripheral intravenous catheter) within one hour of thaw at a dose of 50 million (as available in aliquot) viable TNCs/ kg body weight. The exact dose will depend on the number of viable TNCs available in the final cell product. The volume of cell infusion(s) will be 10 mL/kg, and cell infusion will occur over one hour undertaken in the neonatal unit or the clinical trials facility of Monash Children's Hospital. The custody of care of the product will be transferred from BMDI Cord Blood Bank to the shipper to Monash Health Translation Precinct's Cell Therapy and Regenerative Medicine Platform to the clinical team using appropriate standard operating procedures.

- 1. ***Statistical analysis***

Descriptive statistics will be used to report demographics and outcomes. The categorical variables will be described as frequency and percentage and continuous variables as median (interquartile range) and mean (standard deviation), depending on the normality of the data, which will be determined by the Shapiro-Wilk test. The data will be analysed using STATA Version 17.0 (StataCorp LLC, College Station, TX, USA). Paired t-tests or Wilcoxon signed-rank tests will be performed to compare the laboratory parameters before and after infusion.

- 1. **Safety monitoring and reporting**
     1. Monitoring **(Figure 1 and Table 1):**
        1. The following will be monitored during and within 48 hours of infusion.
     - During infusion
       - Local site reaction (erythema, oedema, extravasation at site of peripheral intravenous catheter site).
       - Any sustained change of 30% or more from baseline in vital signs (HR, RR, BP, SpO2, Temperature).
     - Within the first 48 hours of infusion
       - Any event requiring cardiopulmonary resuscitation.
       - Escalation of respiratory support (intubation of an infant receiving non-invasive respiratory support; or change to high-frequency oscillatory ventilation in an infant receiving conventional ventilation at the time of UCBC infusion).
       - Fluid bolus or initiation/escalation of inotropic support.
       1. Infants will also be monitored clinically for infection within 48 hours of UCBC infusion. Appropriate tests will be performed to diagnose infection (culture-proven bacterial, fungal, viral infection, culture-negative, clinically suspected infection).
       2. Infants will be monitored for features of acute GVHD in the first three months after cell infusion, which includes
          1. Skin involvement: Any erythematous rash with or without skin peeling
          2. Gastrointestinal involvement: vomiting and diarrhoea
          3. Liver involvement: jaundice, conjugated hyperbilirubinemia, and abnormal liver function tests (LFTs).
       3. Physical examinations will be performed before the infusion. Further examinations will be performed at 1, 2, 3, 6, 9, 12, 18 and 24 months post-infusion. In the physical examinations, infants will be evaluated for features of chronic GVHD, such as

Skin involvement: rash, dryness, itching, thickening, change of colour, and temperature intolerance

Nail involvement: nail loss, brittle nails, abnormal texture

Hair involvement: loss of body or scalp hair, grey hair

Mouth involvement: dryness, sensitivity, ulcers, gum disease, tooth decay

Gastrointestinal and liver involvement: loss of appetite, nausea, vomiting, diarrhoea, abdominal distension, abdominal pain, jaundice, abnormal LFTs

Lung involvement: persistent cough, wheezing, breathing difficulties

Musculoskeletal involvement: muscle weakness and cramps, joint stiffness

Genitalia involvement: dryness, ulceration, itching, scarring, narrowing of orifices

- - - 1. The following neurodevelopmental assessments will be performed.

At term-equivalent age: General movements assessment and Hammersmith neonatal neurological examination

At three months corrected age: General movements assessment and Hammersmith infant neurological examination

At 12 months of corrected age: Gross Motor Function Measure-66 and Infant–toddler Social and Emotional Assessment

At two years of corrected age: Bayley Scales of Infant and Toddler development-IV, and medical assessment for disability

- - - 1. Relevant laboratory tests will be performed to monitor the response and to detect any adverse events, such as GVHD

Before the infusion: Cytokine analysis, including interleukin 1b (IL-1b), IL-6, Tumour Necrosis Factor (TNF-α) and IL-10

24 h after the infusion: Cytokine analysis, full blood examination (FBE), chimerism assessment and LFTs

4 day, 1 week and 1 month chimerism assessment

1 week, 1 month and 3 months post-transfusion: FBE and LFTs

Chimerism assessment will be repeated at 3 months if there are detectable donor cells at 1 month testing (optional assessment)

Lymphocyte subset analysis will also be performed if white cell count is low, which is consistent with GVHD

- - 1. **Data Safety Monitoring Board (DSMB)**: A DSMB consisting of a neonatologist, and paediatric neurologist or cell therapist or haemato-oncologist will be established to review all the adverse events. All serious events will be reported to DSMB within 7 days of occurrence.
       - 1. *Interim analysis:* DSMB will be requested to conduct an interim analysis following the enrolment of five patients in each group to assess the feasibility and adverse events. DSMB and HREC will also review whenever there is a serious event to ensure it is safe to continue the trial.
         2. *Criteria for premature termination of trial*: DSMB and HREC will be consulted for an independent review of trial conduct, progress and any serious events related to cell therapy, which will further determine whether the trial requires premature termination. No predetermined interim analysis will be conducted. The analysis will only be conducted after at least 10 infants have been recruited in each stratum.
    2. Reporting:
       1. Any events occurring after the allogeneic UCBC therapy will be reported to the relevant parties. Events will be categorised as per the National Health and Medical Research Council 2016 recommendation^31^ and report any related or unrelated events

**Adverse event (AE)**: Any untoward medical occurrence in a patient or clinical trial participant administered a medicinal product and that does not necessarily have a causal relationship with this treatment.

**Adverse Reaction (AR)**: Any untoward and unintended response to an investigational medicinal product related to any dose administered.

**Serious AE/AR (SAE/SAR)**: Any adverse event/adverse reaction that results in death, is life-threatening, requires hospitalization or prolongation of existing hospitalization, or results in persistent or significant disability or incapacity.

**Suspected Unexpected Serious Adverse Reaction:** An adverse reaction that is both serious and unexpected.

- - - 1. **HREC**: Any SAE/SAR will be reported to the DSMB and HREC within 7 days of occurrence Protocol amendments, annual research progress and safety reports will be submitted to the HREC as needed. Any protocol deviations and breaches will be investigated by the principal investigator and shall be reported to the HREC.
  1. **Data management**
     1. Data collection: The data from electronic medical records will be recorded on a paper case reporting form OR electronic database (REDCAP). The paper records will be stored in a locked filing cabinet, and the electronic records will be secured with secure password protection.
     2. Data retention: The records will be retained securely for 25 years following completion of the study as per Health Records Act 2001, VIC, Act Number 2/2001.
  2. **Participants' reimbursement**: Costs relevant to this trial will be reimbursed. This includes travel for treatment or follow-up, accommodation and meals expenses, and parking costs. All trial treatments, including hospital admission, are provided free of charge.
  3. **Financial disclosure and conflicts of interest:** Dr Razak receives a doctoral scholarship from Monash University and Lions Cord Blood Foundation. Profs Miller and Malhotra receive funding from the National Health and Medical Research Council of Australia. The study will also be supported by funds from the Monash Health Foundation. The funding bodies have no role in the design of the study. They will also have no role in data collection, analysis, and interpretation of data and dissemination of the results.
  4. **Dissemination**: The study design and findings will be disseminated via scientific conferences, publication in peer-reviewed journals, and social media platforms. No identifiable participant data will be reported. Participant families will each receive a summary letter of study outcomes or the publication at the conclusion of the study if desired.

**Figure 1:** Study Flow Chart

**Eligibility** (severe brain injury in preterm)

**Consent**

**Blood** for HLA-typing/matching

**4/6-5/6 HLA-**matched UCBCs availability (***FEASIBILITY***)

Re-confirm **eligibility**

**INTERVENTION (Examine**, **blood** for cytokines, **administer UCBCs)**

**Blood** for cytokines (24 h) & assess for side effects (up to 48 h)

**Blood** for chimerism (4d)

**Blood** for FBE, LFTs and chimerism (1w, 1m)

**GVHD assessment** at 2 m

**Blood** (FBE/LFTs), & **GVHD assessment** (3m)(***SAFETY***)

**MRI** at TEA

**Assessments: Neurological** (3, 12, 24 m) and **GVHD** (6, 9, 12, 18, 24m)

**Table 1**: Safety, adverse events, and neurological outcome monitoring

| Timeline | Cardiorespiratory monitoring | Infection monitoring | Physical examination | Cytokine analysis | FBE*, chimerism assessment and LFT | Neurodevelopmental assessment | Neuroimaging |
| --- | --- | --- | --- | --- | --- | --- | --- |
| -2 to 0 h | √ | √ | √ | √ |  |  |  |
| 0 to 48 h | √ | √ |  |  |  |  |  |
| 24 h |  |  | √ | √ | √ |  |  |
| 4 d |  |  |  |  | √ (Chimerism only) |  |  |
| 7 d |  |  | √ |  | √ |  |  |
| 1 m |  |  | √ |  | √ |  |  |
| 2 m |  |  | √ |  |  |  |  |
| 3 m |  |  | √ |  | √ (Chimersim is optional) | √ (TEA) | √ (MRI at TEA) |
| 6 m |  |  | √ |  |  | √ (3 m CA) |  |
| 9 m |  |  | √ |  |  |  |  |
| 12 m |  |  | √ |  |  | √ (12 m CA) |  |
| 18 m |  |  | √ |  |  |  |  |
| 24 m |  |  | √ |  |  | √ (24 m CA) |  |

**Note**: CA, corrected age; FBE, full blood examination (*-lymphocyte subset analysis for patients with abnormal FBE consistent with GVHD, and chimerism analysis will be repeated at 3, 6 or 12 months if there are detectable donor cells at 1 and/or 3 months testing); LFT, liver function test; MRI, magnetic resonance imaging; TEA, term equivalent age

**References**

1. Yeo KT, Thomas R, Chow SS, et al. Improving incidence trends of severe intraventricular haemorrhages in preterm infants <32 weeks gestation: a cohort study. *Arch Dis Child Fetal Neonatal Ed.* 2020;105(2):145-150.

2. Bolisetty S, Dhawan A, Abdel-Latif M, et al. Intraventricular hemorrhage and neurodevelopmental outcomes in extreme preterm infants. *Pediatrics.* 2014;133(1):55-62.

3. Zhou L, McDonald CA, Yawno T, et al. Feasibility of cord blood collection for autologous cell therapy applications in extremely preterm infants. *Cytotherapy.* 2023.

4. Zhou L, McDonald C, Yawno T, Jenkin G, Miller S, Malhotra A. Umbilical Cord Blood and Cord Tissue-Derived Cell Therapies for Neonatal Morbidities: Current Status and Future Challenges. *Stem Cells Transl Med.* 2022;11(2):135-145.

5. Ahn SY, Chang YS, Sung SI, Park WS. Mesenchymal Stem Cells for Severe Intraventricular Hemorrhage in Preterm Infants: Phase I Dose-Escalation Clinical Trial. *Stem Cells Transl Med.* 2018;7(12):847-856.

6. Bozkaya D, Ceran B, Ozmen E, et al. A New Hope in the Treatment of Intraventricular Haemorrhage in Preterm Infants: Mesenchymal Stem Cells. *Turk Neurosurg.* 2022;32(2):344-346.

7. Paton MCB, McDonald CA, Allison BJ, Fahey MC, Jenkin G, Miller SL. Perinatal Brain Injury As a Consequence of Preterm Birth and Intrauterine Inflammation: Designing Targeted Stem Cell Therapies. *Front Neurosci.* 2017;11:200.

8. McDonald CA, Fahey MC, Jenkin G, Miller SL. Umbilical cord blood cells for treatment of cerebral palsy; timing and treatment options. *Pediatr Res.* 2018;83(1-2):333-344.

9. Nguyen T, Purcell E, Smith MJ, et al. Umbilical cord blood-derived cell therapy for perinatal brain injury: a systematic review & meta-analysis of preclinical studies. *International Journal of Molecular Sciences.* 2023;24(5):4351.

10. Purcell E, Nguyen T, Smith M, et al. Factors Influencing the Efficacy of Umbilical Cord Blood-Derived Cell Therapy for Perinatal Brain Injury. *Stem Cells Transl Med.* 2023.

11. Novak I, Morgan C, Fahey M, et al. State of the Evidence Traffic Lights 2019: Systematic Review of Interventions for Preventing and Treating Children with Cerebral Palsy. *Curr Neurol Neurosci Rep.* 2020;20(2):3.

12. Paton MCB, Wall DA, Elwood N, et al. Safety of allogeneic umbilical cord blood infusions for the treatment of neurological conditions: a systematic review of clinical studies. *Cytotherapy.* 2022;24(1):2-9.

13. Wysoczynski M, Khan A, Bolli R. New Paradigms in Cell Therapy: Repeated Dosing, Intravenous Delivery, Immunomodulatory Actions, and New Cell Types. *Circ Res.* 2018;123(2):138-158.

14. Bae SH, Kong TH, Lee HS, et al. Long-lasting paracrine effects of human cord blood cells on damaged neocortex in an animal model of cerebral palsy. *Cell Transplant.* 2012;21(11):2497-2515.

15. Crompton K, Godler DE, Ling L, et al. Umbilical cord blood cell clearance post-infusion in immune competent children with cerebral palsy. *Cells Tissues Organs.* 2022.

16. Reen DJ. Activation and functional capacity of human neonatal CD4 T-cells. *Vaccine.* 1998;16(14-15):1401-1408.

17. Canto E, Rodriguez-Sanchez JL, Vidal S. Distinctive response of naive lymphocytes from cord blood to primary activation via TCR. *J Leukoc Biol.* 2003;74(6):998-1007.

18. Chen L, Cohen AC, Lewis DB. Impaired allogeneic activation and T-helper 1 differentiation of human cord blood naive CD4 T cells. *Biol Blood Marrow Transplant.* 2006;12(2):160-171.

19. Sorg RV, Kogler G, Wernet P. Identification of cord blood dendritic cells as an immature CD11c- population. *Blood.* 1999;93(7):2302-2307.

20. Nitsche A, Zhang M, Clauss T, Siegert W, Brune K, Pahl A. Cytokine profiles of cord and adult blood leukocytes: differences in expression are due to differences in expression and activation of transcription factors. *BMC Immunol.* 2007;8:18.

21. Risdon G, Gaddy J, Broxmeyer HE. Allogeneic responses of human umbilical cord blood. *Blood Cells.* 1994;20(2-3):566-570; discussion 571-562.

22. Risdon G, Gaddy J, Stehman FB, Broxmeyer HE. Proliferative and cytotoxic responses of human cord blood T lymphocytes following allogeneic stimulation. *Cell Immunol.* 1994;154(1):14-24.

23. Barker JN, Davies SM, DeFor T, Ramsay NK, Weisdorf DJ, Wagner JE. Survival after transplantation of unrelated donor umbilical cord blood is comparable to that of human leukocyte antigen-matched unrelated donor bone marrow: results of a matched-pair analysis. *Blood.* 2001;97(10):2957-2961.

24. Romano M, Fanelli G, Albany CJ, Giganti G, Lombardi G. Past, Present, and Future of Regulatory T Cell Therapy in Transplantation and Autoimmunity. *Front Immunol.* 2019;10:43.

25. Bernardo ME, Fibbe WE. Mesenchymal stromal cells: sensors and switchers of inflammation. *Cell Stem Cell.* 2013;13(4):392-402.

26. Escolar ML, Poe MD, Provenzale JM, et al. Transplantation of umbilical-cord blood in babies with infantile Krabbe's disease. *N Engl J Med.* 2005;352(20):2069-2081.

27. Australia) TNHaMRCtARCaU. National Statement on Ethical Conduct in Human Research. National Health and Medical Research Council. <https://www.nhmrc.gov.au/about-us/publications/national-statement-ethical-conduct-human-research-2007-updated-2018#block-views-block-file-attachments-content-block-1>. Published 2007 (Updated 2018). Accessed 13 March 2023.

28. Papile LA, Burstein J, Burstein R, Koffler H. Incidence and evolution of subependymal and intraventricular hemorrhage: a study of infants with birth weights less than 1,500 gm. *J Pediatr.* 1978;92(4):529-534.

29. de Vries LS, Eken P, Dubowitz LM. The spectrum of leukomalacia using cranial ultrasound. *Behav Brain Res.* 1992;49(1):1-6.

30. Li J, Yawno T, Sutherland A, et al. Preterm white matter brain injury is prevented by early administration of umbilical cord blood cells. *Exp Neurol.* 2016;283(Pt A):179-187.

31. (2016) NHMRC. Guidance: Safety monitoring and reporting in clinical trials involving therapeutic goods. National Health and Medical Research Council. <https://www.nhmrc.gov.au/guidelines-publications/eh59>. Published 2016. Accessed 13 March, 2023.

**Participant Information Sheet/Consent Form – Person/Responsible**

| Title | Safety and Feasibility of Allogeneic Cord Blood-Derived Cell Therapy in Preterm Infants with Severe Brain Injury |
| --- | --- |
| Short Title | ALLO Trial |
| Protocol Number | [2.2] |
| Project Sponsor | Monash Health |
| Coordinating Principal Investigator/ Principal Investigator | Associate Professor Atul Malhotra |
| Associate Investigator(s) | Dr Abdul Razak, Dr Michelle Martin, Dr Lindsay Zhou, Dr Madison Paton, Associate Professor Ngaire Elwood, Dr Courtney McDonald, Professor Suzanne Miller, Professor Rod Hunt |
| Location | Monash Children's Hospital |

**Part 1 What does participation involve?**

**1 Introduction**

We invite you and your baby to participate in this research study because ultrasound scans

have shown that your baby has a severe form of preterm brain injury. The research project is testing a new treatment for severe brain injury. The new treatment is called allogeneic cord blood-derived cell therapy.

This Participant Information Sheet/Consent Form tells you about the research project. It explains the tests and treatments involved. Knowing what is involved will help you decide if you want your baby to participate in the research.

Please read this information carefully. Ask questions about anything you don't understand or want to know more about. Before deciding whether or not your baby can take part, you might want to talk about it with a relative, friend or the your local doctor.

Participation in this research is completely voluntary. If you don't wish your baby to participate, they don’t have to. They will receive the best possible care whether or not they take part.

If you want your baby to participate in the research project, you will be asked to sign the consent section. By signing it, you are telling us that you:

• Understand what you have read

• Consent to your baby taking part in the research project

• Consent to your baby having the tests and treatments that are described

• Consent to using your baby's personal and health information as described.

You will be given a copy of this Participant Information and Consent Form to keep

**2 What is the purpose of this research?**

Severe intraventricular haemorrhage and cystic periventricular leucomalacia are severe forms of brain injuries that can occur in premature babies. Severe intraventricular haemorrhage is swelling of the brain's fluid-filled spaces or damage to the nearby brain tissue due to bleeding. Whereas, periventricular leucomalacia is a type of white matter injury in the brain which leads to the formation of fluid-filled sacs near the brain's fluid-filled spaces due to bleeding, lack of blood supply, infection or inflammation. These babies are at increased risk of death, and those who survive are at high risk of long-term developmental problems, such as cerebral palsy, behavioural or learning difficulties.

Emerging evidence suggests that cell therapies may prevent or reduce the severity of brain injury following intraventricular haemorrhage and periventricular leukomalacia and so decrease the risk of developmental disability. In this study, umbilical cord blood-derived cells (UCBCs) collected and stored from other babies' cord blood will be used for the experimental therapy.

In research studies, UCBCs have already been given to newborn babies with brain injury worldwide to test their safety, feasibility and effectiveness. Our group (led by A/Prof Malhotra) is conducting one such trial using baby’s own cord blood derived UCBCs in extremely preterm babies (born less than 28 weeks of pregnancy) at Monash Children's Hospital.

UCBC therapy is still considered an 'experimental' treatment for babies with brain injury.

This study aims to test whether we can sufficiently match your baby's blood with the cord blood of other babies (which is in long term storage) and whether it is safe to give cord blood-derived cells from other babies to your baby. This is an initial (pilot) study assessing the safety and feasibility of UCBC in preterm babies with severe brain injury. Future large studies may be conducted to test if this therapy improves long-term outcomes.

This research has been initiated by the research team led by A/Prof Atul Malhotra. This team and the other investigators listed above are experienced in using this type of cell therapy.

The study doctor Abdul Razak will use the results of this research to obtain a Doctoral degree in philosophy.

The studies will be conducted at Monash Newborn, Monash Children's Hospital (Monash Health) in partnership with BMDI Cord Blood Bank and Hudson Institute of Medical Research. Matched UCBCs will be obtained by the BMDI Cord Blood Bank, which the Hudson Institute of Medical Research will further process before giving it to your baby.

This research is being conducted by Monash Health and funded by NHMRC, Monash Health Foundation, and the Lions Cord Blood Foundation.

**3 What does participation in this research involve?**

Participation will involve:

1. Blood processing and matching (BMDI Cord Blood Bank)
2. Final preparation of cell product and giving cord blood cells to baby (Hudson Institute of Medical Research and Monash Health)
3. Blood tests in the baby (Monash Health)
4. Clinical examination (Monash Health)

Your baby's routine brain scans (ultrasound) during the first few weeks of life have confirmed the diagnosis of severe brain injury, which makes your baby eligible for the trial. If you agree for your baby to participate, a blood test will be done to 4/6 HLA (66%) match your baby's blood with the cord blood unit available at BMDI Cord Blood Bank. If a match is successful, the UCBCs will be obtained from the BMDI Cord Blood Bank a day or two before the infusion, and processed at the Hudson Institute of Medical Research before giving it to your baby.

A maximum of twenty babies with severe brain injury are planned to receive UCBCs in this study.

UCBCs will be given while your baby is in the hospital via a drip and will take up to 1 hour to give. Your baby may require the insertion of a new 'drip' for this infusion. The process is similar to having a blood transfusion. We will monitor your baby closely for any side effects during the infusion while the baby remains hospitalised in the nursery for the first 48 hours. We will also continue to follow your baby's progress, including neurodevelopment and check for any side effects related to the treatment until the baby is two years old. This includes monthly examinations for the first three months, then once every three months for the remainder of the first year, and then twice in the second year (see the end of the document for figure 1).

We will perform blood tests to check how the UCBCs affect your baby's immune response, whether the baby's body is accepting or rejecting UCBCs, and any side effects related to the therapy. This involves taking a small amount of blood, 0.5-1 mL, just before the infusion, and 24 h, 4 days, 7 days, 1 month, and in some cases 3 months after infusion, which in total will be less than a teaspoon.

No additional costs are associated with participation in this trial, nor will you or your baby be paid. All medications, tests and medical care required for the trial will be provided to your baby free of charge.

You and your baby may be reimbursed for any reasonable travel, and parking associated with the research project follow-up visits, which are not part of routine follow-up.

**4 What does the baby have to do?**

There are no restrictions for ongoing treatment for your baby to participate in the study. All appropriate medications or treatments required for the baby can be continued without restrictions.

**5 Other relevant information about the research project**

There may be up to 20 babies taking part in this study at Monash Children's Hospital. No babies from other hospitals will be enrolled. Only eligible babies receiving experimental therapy will be involved with no control group. **Table 1** highlights the monitoring details.

**Table 1**: Safety, adverse events, and neurological outcome monitoring

| Timeline | Cardiorespiratory monitoring | Infection monitoring | Physical examination | Cytokine analysis | FBE*, chimerism assessment and LFT | Neurodevelopmental assessment | Neuroimaging |
| --- | --- | --- | --- | --- | --- | --- | --- |
| -2 to 0 h | √ | √ | √ | √ |  |  |  |
| 0 to 48 h | √ | √ |  |  |  |  |  |
| 24 h |  |  | √ | √ | √ |  |  |
| 4 d |  |  |  |  | √ chimerism only |  |  |
| 7 d |  |  | √ |  | √ |  |  |
| 1 m |  |  | √ |  | √ |  |  |
| 2 m |  |  | √ |  |  |  |  |
| 3 m |  |  | √ |  | √ (chimerism is optional) | √ (TEA) | √ (MRI at TEA) |
| 6 m |  |  | √ |  |  | √ (3 m CA) |  |
| 9 m |  |  | √ |  |  |  |  |
| 12 m |  |  | √ |  |  | √ (12 m CA) |  |
| 18 m |  |  | √ |  |  |  |  |
| 24 m |  |  | √ |  |  | √ (24 m CA) |  |

Note: CA, corrected age; FBE, full blood examination (*-lymphocyte subset analysis for patients with abnormal FBE consistent with GVHD, and chimerism analysis will be repeated at 3, 6 or 12 months if there are detectable donor cells at 1 and or 3 months testing); LFT, liver function test; MRI, magnetic resonance imaging; TEA, term equivalent age

**6 Does the baby have to take part in this research project?**

Participation in any research project is completely voluntary. If you do not wish for your baby to participate, the baby does not have to. If you decide that your baby can take part and later change your mind, you can withdraw the baby from the project at any stage.

If you do decide that your baby can take part, you will be given this Participant Information and Consent Form to sign, and you will be given a copy to keep.

Your decision whether the baby can take part or not, or take part and then be withdrawn, will not affect the baby's routine treatment, your or the baby's relationship with those treating them, or the baby's relationship with Monash Children's Hospital.

**7 What are the alternatives to participation?**

Your baby does not have to participate in this research project to receive treatment at this hospital. Your baby's routine care will not change if you decide not to participate in this project.

**8 What are the possible benefits of taking part?**

We do not yet know whether the chances (or severity) of your baby developing long-term effects

of brain injury will be reduced by the UCBCs. This study is being done mainly to assess the feasibility of obtaining a match, and safety of administration of single infusion of UCBCs in babies with this brain condition. We will use the results of this trial to plan larger studies that will explore if UCBCs are effective for this type of brain injury.

**9 What are the possible risks and disadvantages of taking part?**

Few studies have shown some safety data using closely matched donor UCBCs for babies with severe brain injury and other conditions, but more studies are required. There may be unknown risks associated with participation.

All medical treatments can have side effects; therefore, we will carefully monitor your baby. Given that the cells are from the different babies' blood, there is a risk of rejection of cells and the possibility of graft-versus-host disease. The other potential risks, though less likely, include infusion-related reactions, including anaphylaxis, local site reactions, infection, and unforeseen reactions. These risks are explained in more detail below.

**Graft-versus-host disease (GVHD)**

Graft-versus-host disease (GvHD) is a possible complication that can occur when the donated UCBCs react against the baby's body cells. This happens because the UCBCs see the baby's cells as foreign and attack them. The exact risk of GvHD following UCBC therapy is uncertain, but we will closely monitor it to keep the baby safe.

GvHD can affect different parts of the baby's body, like the skin, gut, and liver. This can cause problems such as rash, diarrhoea, and jaundice. GvHD can also affect other areas like the mouth, eyes, lungs, genitals, and joints. The severity of GvHD can range from mild to severe, and it may last for months or even years. In rare cases, it may lead to death.

To reduce the risk of GvHD, we have taken several steps. We are using cells from umbilical cord blood, which are only used once. Cord blood derived from healthy-term pregnancies is known to have a lower risk of GvHD as compared to blood derived from adult donors due to the immaturity of cord blood cells. We will also match the baby's human leukocyte antigen (blood cell fingerprint) with the donor's cells, and we are not using strong treatments (like those used in cancer) that may damage the immune system. However, it's important to know that these steps cannot completely eliminate the risk. A previous study using similar therapy in preterm babies found no cases of GvHD in the babies they followed up.

Also, we don't have enough information about the severity of GvHD following UCBCs in preterm babies. It can vary in its effects and, in some cases, be life-threatening. But a study found low-severity GvHD in babies treated with UCBCs in the neonatal age group, and it was manageable.

We have a team of blood and cancer specialists involved in the research who will closely monitor the baby's condition for any symptoms. They will take the necessary steps to manage any potential risks. If you would like to talk with them to learn more about this condition and how it can be managed, we can arrange that for you.

**Local site reactions**

Whenever a drug or infusion is given via a drip, there is potential for a local reaction - redness or swelling at the infusion site.

**Infusion-related reactions**

This describes a reaction including anaphylaxis (a serious allergic reaction) that may cause your baby to have changes in their vital signs such as heart rate, blood pressure and oxygen levels.

If this were to occur, the infusion would be stopped, and standard medications would be given to treat the allergy to stabilise your baby.

**Transmission of infection**

The BMDI Cord Blood Bank collects cord blood from healthy baby donors for cell therapy applications or transplantation. Many procedures are in place to ensure that the UCBCs collected for these applications are very safe. Once collected, UCBCs are thoroughly tested for infections and viruses, including Hepatitis B and C, Syphilis, T-cell Lymphocytic Virus and the Human Immunodeficiency Virus. If there are any problems identified with the donated cord blood, it is destroyed. The risk of transferring infections cannot be ruled out despite screening all blood donations. Apart from these infections, there is also a possibility of other infections that have not been tested or infections occurring from contamination while the UCBCs are being processed for storage and infusion. Whilst preparing the cells, we follow careful procedures to check that the cells have no infection.

**Risks from the intravenous catheter and blood tests**

An intravenous cannula inserted or blood taken may cause discomfort, bruising, minor infection or bleeding. We will try to keep your baby as comfortable as possible during these procedures and minimise the risk of bruising, bleeding or infection. If this happens, it can be easily treated.

**10 What will happen to the baby's test samples?**

Samples of your baby's blood obtained during this study for laboratory analysis will be stored and analysed in Monash Health and Monash Health's Translational Research Precinct, Clayton. Your baby's blood samples collected for this part of the project will be de-identified at the collection and allocated a study code. Any information identifying your baby, such as name, address, date of birth and hospital record number, will be removed before your baby's blood sample goes to the laboratory.

We expect all the blood we collect from your baby will be used up during cell processing and laboratory testing. However, if there is any blood or cells left over, they will be discarded unless you agree to provide specific consent to be used for later research following Human Research Ethics approval.

**11 What if new information arises during this research project?**

Sometimes during the course of a research project, new information becomes available about the treatment that is being studied. If this happens, the study doctor will tell you about it and discuss whether you want your baby to continue in the research project. If you decide that your baby can continue in the research project, you may be asked to sign an updated consent form.

Also, upon receiving new information, the study doctor might consider it in your baby's best interest to withdraw them from the research project. If this happens, the doctor will explain the reasons.

**12 Can the baby have other treatments during this research project?**

There are no contraindications to any treatment during this research project. Your baby can receive other treatments.

**13 What if I withdraw my baby from this research project?**

If you decide to withdraw the baby from the project, please notify the research team member before you withdraw them. This notice will allow that person or the research supervisor to discuss any health risks or special requirements linked to withdrawing.

If you do withdraw the baby during the research project, the study doctor and relevant study staff will not collect additional personal information from the baby, although personal information already collected will be retained to ensure that the results of the research project can be measured properly and to comply with the law. You should be aware that data collected by the team until you withdraw the baby will form part of the research project results. If you do not want them to do this, you must tell them before you join the research project.

**14 Could this research project be stopped unexpectedly?**

This research project aims at assessing the safety and feasibility of the treatment, and may be stopped unexpectedly for reasons such as:

• Unacceptable serious side effects

• Decision by local regulatory/health authorities due to new information

**15 What happens when the research project ends?**

Please let the research team know if you want to know about the study's results. When the study results have been analysed and published, we can send you a copy of the publication. Your baby's privacy will be protected and will remain anonymous in the results.

**Part 2 How is the research project being conducted?**

**16 What will happen to information about the baby?**

By signing the consent form, you consent to the study doctor and relevant research staff to collect and use personal information about your baby for the research project. Information about your baby may be obtained from their health records held at this and other health service(s) for the purpose of this research. Any information obtained in connection with this research study that can identify you or your baby will remain confidential and will only be used for the purpose of this research study. It will only be disclosed with your permission, except as required by law.

It is anticipated that the results of this research project will be published and presented in various forums. In any publication or presentation, information will be provided in such a way that your baby cannot be identified. The records for this study will be kept in a locked filing cabinet, and the computer records will only be accessible by a password known to the research team. Once the information has been collected, it will be stored with a study number but not your baby's name. Information is stored for 25 years in accordance with Victorian law and, after this, will be disposed of confidentially.

Information about your baby's participation in this research project will be recorded in their health records.

In accordance with relevant Australian and/or Victorian privacy and other relevant laws, you have the right to request access to your baby's information collected and stored by the study team. You also have the right to request that any information with which you disagree be corrected. Please contact the study team member at the end of this document if you want to access your baby's information.

**17 Complaints and Compensation**

If the baby suffers any injuries or complications from this research project, you should contact the study team as soon as possible, and you will be assisted with arranging appropriate medical treatment for the baby. If the baby is eligible for Medicare, they can receive any medical treatment required to treat the injury or complication, free of charge, as a public patient in any Australian public hospital.

**18 Who is organising and funding the research?**

The research team led by A/Prof Atul Malhotra is conducting this research project. This project is funded by research funds from NHMRC, Monash Health Foundation, and the Lions Cord Blood Foundation, which also provides a scholarship to the study doctor Abdul Razak. Dr Abdul Razak also receives a scholarship from Monash University. BMDI Cord Blood Bank is performing the storage and release of UCBCs, and Hudson Institute of Medical Research is formulating the final cell product.

**19 Who has reviewed the research project?**

All research in Australia involving humans is reviewed by an independent group called the Human Research Ethics Committee (HREC). The ethical aspects of this research project have been approved by Monash Health HREC.

This project will be carried out according to the *National Statement on Ethical Conduct in Human Research (2018)*. This statement has been developed to protect the interests of people who agree to participate in human research studies.

**20 Further information and who to contact**

If you want any further information concerning this project or if the baby has any medical problems which may be related to their involvement in the project (for example, any side effects), you can contact the principal study doctor or any of the following people:

**Clinical contact persons**

| Name | A/Prof Atul Malhotra |
| --- | --- |
| Position | Consultant Neonatologist, Monash Children's Hospital |
| Telephone | 03 8572 3650 |
| Email | [Atul.malhotra@monash.edu](mailto:Atul.malhotra@monash.edu) |

| Name | Dr Abdul Razak |
| --- | --- |
| Position | Neonatal Fellow, Monash Children's Hospital |
| Telephone | 03 8572 3650 |
| Email | Abdul.razak@monash.edu |

If you have any complaints about any aspect of the project, the way it is being conducted or any questions about being a research participant in general, then you may contact:

**Reviewing HREC approving this research** **and HREC Executive Officer details**

| Name | Monash Health Human Research Ethics Committee |
| --- | --- |
| Position | HREC Executive Officer |
| Telephone | 03 9594 4611 |
| Email | Research@monashhealth.org |

**Consent Form – Person Responsible**

| Title | Safety and Feasibility of Allogeneic Cord Blood-Derived Cell Therapy in Preterm Infants with Severe Brain Injury |
| --- | --- |
| Short Title | ALLO Trial |
| Project Sponsor | Monash Health |
| Coordinating Principal Investigator/ Principal Investigator | Associate Professor Atul Malhotra |
| Associate Investigator(s) | Dr Abdul Razak, Dr Michelle Martin, Dr Lindsay Zhou, Dr Madison Paton, Associate Professor Ngaire Elwood, Dr Courtney McDonald, Professor Suzanne Miller, Professor Rod Hunt |
| Location | Monash Children's Hospital |

**Declaration by Person Responsible**

I have read the Participant Information Sheet, or someone has read it to me in a language I understand.

I understand the purposes, procedures and risks of the research described in the project.

I have had an opportunity to ask questions, and I am satisfied with the answers I have received.

I believe that the participation of my baby in this study is not contrary to their best interests.

I freely agree to my baby participating in this research project as described and understand that I am free to withdraw my baby at any time during the research project without affecting their future health care.

I am aware of my responsibilities as the Person Responsible for the baby. I understand that I will be assisting my baby in meeting their responsibilities whilst participating in this study.

I understand that I will be given a signed copy of this document to keep on behalf of my baby.

I give permission for my baby's doctors, other health professionals, hospitals or laboratories outside this hospital to release information to Monash Health concerning the participant's disease and treatment for the purposes of this research project. I understand that such information will remain confidential.

I consent to the storage and use of blood and tissue samples taken from the participant for use, as described in the relevant section of the Participant Information Sheet, for:

This specific research project

PLUS optional consents:

Other research that is closely related to this research project and has received ethics approval

Any future ethically approved research projects

|  |  |  | | | | |  |
| --- | --- | --- | --- | --- | --- | --- | --- |
|  | Name of Baby (please print) |  | | | | |  |
|  |  |  | | | | |  |
|  | Name of Parent/Guardian (please print) | | |  | | |  |
|  |  | | |  | | |  |
|  | Signature of Person Responsible | |  | | Date |  |  |
|  | | | | | | | |

|  | | | | | | | |
| --- | --- | --- | --- | --- | --- | --- | --- |
|  | Name of Witness* to  Person Responsible Signature (please print) | | |  | | |  |
|  |  | |  |  | | |  |
|  | Signature |  | | | Date |  |  |
|  | | | | | | | |

* Witness is not to be the investigator, a member of the study team or their delegate. If an interpreter is used, the interpreter may not act as a witness to the consent process. The witness must be 18 years or older

**Declaration by Study Doctor/Senior Researcher^†^**

I have verbally explained the research project, its procedures and risks, and I believe that the person responsible has understood that explanation.

|  | | | | | | | |
| --- | --- | --- | --- | --- | --- | --- | --- |
|  | Name of Study Doctor/  Senior Researcher^†^ (please print) | |  | | |  | |
|  | | | | | |  | |
|  | Signature |  | | Date |  | |  |
|  | | | | | | | |

^†^ A senior research team member must explain and provide information concerning the research project.

Note: All parties signing the consent section must date their own signature.

**Form for Withdrawal of Participation – Person Responsible**

| Title | Safety and Feasibility of Allogeneic Cord Blood-Derived Cell Therapy in Preterm Infants with Severe Brain Injury |
| --- | --- |
| Short Title | ALLO Trial |
| Project Sponsor | Monash Health |
| Coordinating Principal Investigator/ Principal Investigator | Associate Professor Atul Malhotra |
| Associate Investigator(s) | Dr Abdul Razak, Dr Michelle Martin, Dr Lindsay Zhou, Dr Madison Paton, Associate Professor Ngaire Elwood, Dr Courtney McDonald, Professor Suzanne Miller, Professor Rod Hunt |
| Location | Monash Children's Hospital |

**Declaration by Person Responsible**

I wish to withdraw my baby from participating in the above research project and understand that such withdrawal will not affect the baby's routine treatment, relationship with those treating them or their relationship with Monash Children's Hospital.

|  |  |  | | | | |  |
| --- | --- | --- | --- | --- | --- | --- | --- |
|  | Name of Baby (please print) |  | | | | |  |
|  |  |  | | | | |  |
|  | Name of Parent/Guardian (please print) | | |  | | |  |
|  |  | | |  | | |  |
|  | Signature of Person Responsible | |  | | Date |  |  |
|  | | | | | | | |

|  | | | | | | | |
| --- | --- | --- | --- | --- | --- | --- | --- |
|  | Name of Study Doctor/  Senior Researcher (please print) | |  | | |  | |
|  | | | | | |  | |
|  | Signature |  | | Date |  | |  |
|  | | | | | | | |

**Declaration by Study Doctor/Senior Researcher^†^**

I have verbally explained the research project, its procedures and risks, and I believe that the person responsible has understood that explanation.

|  | | | | | | | |
| --- | --- | --- | --- | --- | --- | --- | --- |
|  | Name of Study Doctor/  Senior Researcher^†^ (please print) | |  | | |  | |
|  | | | | | |  | |
|  | Signature |  | | Date |  | |  |
|  | | | | | | | |

^†^ A senior research team member must explain and provide information concerning the research project.

Note: All parties signing the consent section must date their own signature.

Figure 1: Study flow diagram

**ALLO Trial Case Report Form**

| **Study ID number** |  | |
| --- | --- | --- |
| **Name** | **DOB** | **Gender** M/F |
| **Birth weight (grams)** | **Gestational age at birth (weeks)** | |
| **Antenatal steroids x1 X 2** | **FGR** Y/N | |
| **Antenatal ultrasounds** | | |
| **Pregnancy complications** | | |
| **Mode of delivery** | | |
| **Apgar scores** | | |
| **Resuscitation –** None/Oxygen/CPAP/Mask PPV/Intubation/CPR/Adrenaline  **Resuscitation details** | | |
| **Surfactant/ MIST** | | |
| **Ventilation details** | | |
| **Echo/ PDA** | | |
| **IVH** Severe IVH Y/N  **PVL/ PVE** Cystic PVL Y/N  **Details of cranial ultrasound** | | |
| **Culture proven sepsis** | | |
| **Other diagnoses** | | |
| **Medications** | | |

**Details of care on day of UCBCs**

| **Date** | **Day of life** | | **Corrected gestation** | |
| --- | --- | --- | --- | --- |
| **Current Weight (date)** | | | | |
| **Current respiratory support** | | **Settings** | | **FiO2** |
| **Last cranial USS (date)** | | | | |
| **Last Echocardiogram (date)** | | | | |
| **Last serum chemistry (date)**  **FBE (date)** | | | | |
| **Medications** | | | | |
| **Feeds** | | | | |

**Vascular access, site**

**Time of administration (24h clock)**

**Volume of infusion (mL): Cell dose:**

**Monitoring on day of UCBC administration**

**Events (including investigations) in the first 48 hours**

**Local site** – Erythema/ Swelling/ Any other change_____________________

**Respiratory/ Cardiovascular**

| **Gas** | **pH** | **pCO2** | | **pO2** | **BE** | **HCO3** | **Lactate** | **OI** | **RSS** |
| --- | --- | --- | --- | --- | --- | --- | --- | --- | --- |
| **-1h** |  |  | |  |  |  |  |  |  |
| **Settings** |  | | | **MAP** |  | **FiO2** |  |  | |
| **Acceptable** |  | |  |  |  |  |  |  |  |
| **1h** |  |  | |  |  |  |  |  |  |
| **Settings** |  | | | **MAP** |  | **FiO2** |  |  | |
| **6h** |  |  | |  |  |  |  |  |  |
| **Settings** |  | | | **MAP** |  | **FiO2** |  |  |  |
| **Cessation of acceptable limits for gas** | | | | | | | | | |
| **12h** |  |  | |  |  |  |  |  |  |
| **Settings** |  | | | **MAP** |  | **FiO2** |  |  |  |
| **24h** |  |  | |  |  |  |  |  |  |
| **Settings** |  | | | **MAP** |  | **FiO2** |  |  | |
| **48 h** |  |  | |  |  |  |  |  |  |
| **Settings** |  | | | **MAP** |  | **FiO2** |  |  |  |

| **Time**  **(hrs)** | **-2** | **-1.75** | **-1.5** | **-1.25** | **-1** | **-0.75** | **-0.5** | **-0.25** | **0** | **Accept-able** |
| --- | --- | --- | --- | --- | --- | --- | --- | --- | --- | --- |
| **BP** |  |  |  |  |  |  |  |  |  |  |
| **HR** |  |  |  |  |  |  |  |  |  |  |
| **RR** |  |  |  |  |  |  |  |  |  |  |
| **Temp** |  |  |  |  |  |  |  |  |  |  |

| **Time**  **(hrs)** | **0** | | **.25** | | | | **.5** | | **.75** | | | **1** | | **2** | | **3** | **4** | | **5** | | **6** | | **7** | | **8** | | | **9** |
| --- | --- | --- | --- | --- | --- | --- | --- | --- | --- | --- | --- | --- | --- | --- | --- | --- | --- | --- | --- | --- | --- | --- | --- | --- | --- | --- | --- | --- |
| **BP** |  | | |  | |  | | |  | | |  |  | | |  |  | |  | |  | |  | |  | | |  |
| **HR** |  | | |  | |  | | |  | | |  |  | | |  |  | |  | |  | |  | |  | | |  |
| **RR** |  | | |  | |  | | |  | | |  |  | | |  |  | |  | |  | |  | |  | | |  |
| **Temp** |  | | |  | |  | | |  | | |  |  | | |  |  | |  | |  | |  | |  | | |  |
| **Time**  **(hrs)** | **10** | **11** | | | **12** | | | **13** | | **14** | **15** | | | | **16** | **17** | **18** | **19** | | **20** | | **21** | | **22** | | **23** | **24** | |
| **BP** |  |  | | |  | | |  | |  |  | | | |  |  |  |  | |  | |  | |  | |  |  | |
| **HR** |  |  | | |  | | |  | |  |  | | | |  |  |  |  | |  | |  | |  | |  |  | |
| **RR** |  |  | | |  | | |  | |  |  | | | |  |  |  |  | |  | |  | |  | |  |  | |
| **Temp** |  |  | | |  | | |  | |  |  | | | |  |  |  |  | |  | |  | |  | |  |  | |
| **Time**  **(hrs)** | **27** | **30** | | | **33** | | | **36** | | **39** | **42** | | | | **45** | **48** |  |  | |  | |  | |  | |  |  | |
| **BP** |  |  | | |  | | |  | |  |  | | | |  |  |  |  | |  | |  | |  | |  |  | |
| **HR** |  |  | | |  | | |  | |  |  | | | |  |  |  |  | |  | |  | |  | |  |  | |
| **RR** |  |  | | |  | | |  | |  |  | | | |  |  |  |  | |  | |  | |  | |  |  | |
| **Temp** |  |  | | |  | | |  | |  |  | | | |  |  |  |  | |  | |  | |  | |  |  | |

Comments:

| **Adverse event (including investigations)details** | **AE classification*** |
| --- | --- |
| **Respiratory** |  |
| **Cardiovascular** |  |
| **Haematologic** |  |
| **Renal** |  |
| **Neurological** |  |
| **Infection** |  |
| **Systemic** |  |
| **Other** |  |

*Refer to protocol

**Overall outcomes during nursery stay**

| **Days of ventilation** | **Invasive** | | **Noninvasive** |
| --- | --- | --- | --- |
| **Days to room air** | **Home oxygen**  Y/N | | **Amount** |
| **Length of stay** | | **Referral to NDIS** | |
| **Term MRI brain** | | | |
| **Other morbidities or outcomes relevant to the study before discharge (please give details)**  **FEASIBILITY: Y/N HLA Matching: 4/6, 5/6, 6/6 HLA Loci matched:**  **SAFETY: Y/N**  **Necrotising enterocolitis**  **Culture proven late onset sepsis**  **Bronchopulmonary dysplasia**  **ROP**  **Intraventricular haemorrhage**  **Comments** | | | |
| **Neurodevelopment testing during admission**  **General Movements**  **HINE** | | | |
| **Medications on discharge** | | | |

| **Death before nursery discharge**  **Cause of death** |
| --- |

**GVHD Assessment and bloods**

| **Trial** | **ALLO-Trial** | | | | | | | |
| --- | --- | --- | --- | --- | --- | --- | --- | --- |
| **Patient ID** |  | | | | | | | |
| **Review** | **Time point** | **General condition** | **Laboratory evaluation** | **Skin** | **Liver** | **GIT** | **Chimerism** |  |
| **0** | Pre-infusion |  |  |  |  |  | N/A |  |
| **1** | 24 hours post |  |  |  |  |  |  |  |
| **2** | 1 week |  |  |  |  |  |  |  |
| **3** | 1 month |  |  |  |  |  |  |  |
| **4** | 2 month |  |  |  |  |  |  |  |
| **5** | 3 month |  |  |  |  |  |  |  |
| **6** | 6 month |  |  |  |  |  |  |  |
| **7** | 9 month |  |  |  |  |  |  |  |
| **8** | 12 months |  |  |  |  |  |  |  |
| **9** | 18 months |  |  |  |  |  |  |  |
| **10** | 24 months |  |  |  |  |  |  |  |

**Follow up till 2 years of age**

| ***Term equivalent age (date)*** |
| --- |
| **Physical examination** |
| **Growth parameters** |
| **Development (TEA)**  **GMA**  **HNNE**  **Comments** |

| ***3 months of corrected age(date)*** |
| --- |
| **Physical examination** |
| **Growth parameters** |
| **Development**  **GMA**  **HINE**  **Comments** |

| ***12 months of age(date)*** |
| --- |
| **Physical examination** |
| **Growth parameters** |
| **Development**  **GMFM-66**  **Infant-toddler social emotional assessment**  **Comments** |

| ***24 months of age(date)*** |
| --- |
| **Physical examination** |
| **Growth parameters** |
| **Development (BSID IV)**  **Medical assessment of disability**  **Comments** |

*As clinically indicated

**Appendix**

***Adverse Event Definition:***

*An adverse event (AE) is any untoward medical occurrence in a patient administered a pharmaceutical product and that does not necessarily have a causal relationship with this treatment. An adverse event can therefore be any unfavorable and unintended sign (including an abnormal laboratory finding), symptom, or disease temporally associated with the use of a medicinal product, whether or not related to the medicinal product.* *Pre-existing conditions that worsen after drug administration will be reported as adverse events. Generally an increase/ worsening of pre-existing disease status to more than 30% of baseline are reported as an adverse event. (For eg, a baseline heart rate increase from 150/ minute to more than 195/ minute, an increase of FiO2 requirement from a baseline 50% to more than 65%). This will be predefined and recorded for all infants before the start of cell therapy.*

***Adverse Event Severity and Relationship to hAEC:***

*The severity of AEs will be graded using the National Cancer Institute Common Terminology Criteria for Adverse Events (NCI-CTCAE), v4.0. For AEs that are not identified in the NCI-CTCAE, the following scale will be used:*

*Grade 1 Mild*

*Grade 2 Moderate*

*Grade 3 Severe*

*Grade 4 Life-threatening or disabling*

*Grade 5 Death related to an AE*

*The assessment of causality will be made by the treating physician using the following definitions:*

***Unrelated:***

*This category is applicable to adverse events that are judged to be clearly and incontrovertibly due to extraneous causes (disease, environment, etc.) and do not meet the criteria for drug relationship listed under Unlikely, Possible or Probable.*

***Unlikely:***

*In general, this category is applicable to an adverse event that meets the following criteria (must have the first two):*

*1. It does not follow a reasonable temporal sequence from administration of the drug.*

*2. It may readily have been produced by the patient’s clinical state, environment or toxic factors, or other modes of therapy administered to the patient.*

*3. It does not follow a known pattern of response to the suspected drug.*

*4. It does not reappear or worsen when the drug is re-administered.*

***Possible:***

*This category applies to adverse events for which the connection with hAEC appears unlikely but cannot be ruled out with certainty. An adverse event may be considered possibly related if or when (must have the first two):*

*1. It follows a reasonable temporal sequence from administration of the drug.*

*2. It may have been produced by the patient’s clinical state, environment or toxic factors, or other modes of therapy administered to the patient.*

*3. It follows a known pattern of response to the suspected drug.*

***Probable:***

*This category applies to adverse events that are considered with a high degree of certainty to be related to hAEC. An adverse event may be considered probable, if (must have the first three):*

*1. It follows a reasonable temporal sequence from administration of the drug.*

*2. It cannot be reasonably explained by the known characteristics of the patient’s clinical state, environment or toxic factors, or other modes of therapy administered to the patient.*

*3. It disappears or decreases on cessation or reduction in dose.*

*4. It follows a known pattern of response to the drug.*

*5. It reappears on re-challenge.*

***Serious Adverse Event Definition:***

*A serious adverse event (SAE) is any untoward medical occurrence that occurs at any dose. An AE in a clinical trial is designated to be serious if it results in death, is life-threatening, requires inpatient hospitalisation, or prolongs existing hospitalisation, results in persistent or significant disability or incapacity.*
