# Supplementary material for: Safety and feasibility of allogeneic cord blood-derived cell therapy in preterm infants with severe brain injury (ALLO trial): a phase-1 trial protocol
Source: BMJ Open. 2025 Jun 18;15(6):e100389. doi: 10.1136/bmjopen-2025-100389 (PMC12182109; doi:10.1136/bmjopen-2025-100389)
Supplement: Supplementary file 2 [file bmjopen-2025-100389.reviewer_comments.pdf]

PEER REVIEW HISTORY

BMJ Open publishes all reviews undertaken for accepted manuscripts. Reviewers are asked to complete a checklist review form and are provided with free text boxes to elaborate on their assessment. These free text comments are reproduced below.

ARTICLE DETAILS

Title (Provisional)

Safety and Feasibility of Allogeneic Cord Blood-Derived Cell Therapy in Preterm Infants with Severe Brain Injury (ALLO Trial) – A Phase-1 Trial Protocol

Authors

Razak, Abdul; Connelly, Kristyn; Hunt, Rod W; Miller, Suzanne Lee; McDonald, Courtney A; Jenkin, Graham; Zhou, Lindsay; Paton, Madison Claire Badawy; Martin, Michelle; Liu, Lin; Hart, Cathie; Elwood, Ngaire; Malhotra, Atul

---

VERSION 1 - REVIEW

**Reviewer**	**1**
**Name**	**Wang, Laishuan**
**Affiliation**	**Children's Hospital of Fudan University**
**Date**	**05-May-2025**
**COI**	**None**

Well designed and well prepared trial ptotocol and I would expect good results and will benifit to the severe premature brain injury at last.

**Reviewer**	**2**
**Name**	**Battersby, Cheryl**
**Affiliation**	**Imperial College London, Neonatal Medicine**
**Date**	**23-May-2025**
**COI**	**None**

Thank you for asking me to review this protocol of the important ALLO trial, an open-label phase 1 single -arm feasibility and safety study. The ALLO trial aims to assess the feasibility and safety of partially HLA-matched allogeneic UCBC therapy in preterm infants with severe brain injury. Eligible babies are preterm infants born before 28 weeks (ALLO-1) or between 28 and 36+6 weeks (ALLO-2) gestational age with severe brain injury identified on

neuroimaging. Infusion will occur within two to three weeks of confirmation of eligibility, contingent on clinical stability and absence of active sepsis.

Primary outcomes are safety and feasibility.

I have minor recommendations to the main manuscript to improve clarity to the reader , which I appreciate is a shorter and more succinct version of the very comprehensive attached protocol.

1) Abstract: Primary outcome clarity

Suggest to rephrase the primary outcome in the abstract so Primary outcome is clear, rather than in brackets. Change from "Feasibility is defined as having more than 60% of enrolled infants with an eligible allogeneic cord blood unit available .... (primary outcomes)" to

"Primary outcome includes: i) feasibility, defined as having more than 60% of enrolled infants with an eligible allogeneic cord blood unit available ii) safety defined as the absence of severe adverse events within 48 hours of infusion or graft-versus-host disease within three months of infusion"

2) page 10 "Cell Infusion" duration (one hour) should be included in the intervention section

Abstract

In the "Ethics and Dissemination", rather than "The study complies with the Australian National Statement on Ethical Conduct in Human Research", I would include that there is research approval.

"Monash HREC has granted full ethics approval (RES-23-0000-297A), including approval for the research use of allogeneic cord blood from compassionate donations provided by healthy donors, facilitated through the BMDI Cord Blood Bank, which operates as part of the AusCord network. The committee has endorsed the inclusion of patients with severe brain injury" or something similar/shorter

Main manuscript:

There is no discussion session. I am not sure if this is a requirement, but I do think some of what is in the main protocol, can come into the discussion, example discussion points to highlight include:

1) Expanding on the potential immune risk and potential need for HLA matching in this cohort. Need for more explanation on HLA matching (either in intro or discussion).

2) Justify the 2-3 week period of intervention window with available literature

3) Justify infusion rate - is there evidence this reduces risk of GVHD? or existing literature to suggest this is optimal rate?

4) Maybe discuss more about difficulty of risk- benefit analysis and the need for a feasibility and safety study

5) Also discuss paucity of literature regarding GVHD in neonatal population and the different aims in neonatal cell therapy versus in haematological patients. - that would you expect low levels of GVHD

6) Also important to add what the treatment is if there is GVHD and the implications short and long term of this condition

Minor point about terminology: page 15 and throughout. This may be an Australian term, but I wonder if an alternative term can be used to "consumers" for people with lived experience of neonatal care and /or cerebral palsy. It makes it sound commercial or transactional, which it isn't. This may be a term mandated by the Australian authorities but I would consult PPIE groups to make sure this is acceptable, or just say parents or people with lived experience.

Page 29 flow chart: some of the fonts are too big and can't see the words in the flow chart

Summary:

This is a very important study, and offers potentially new hope for these infants. I commend the researchers for taking the long protracted, but important and necessary in testing this intervention in these high risk populations. The protocol has been well designed and thought through, and ethically sound. My recommendations were focussing more on readability and suggesting some of the detail of the protocol is brought into a discussion section in the main body of this manuscript. Suggest to accept with minor revisions.

VERSION 1 - AUTHOR RESPONSE

Reviewer 1 (Dr. Laishuan Wang) comments:

REVIEWER 1 COMMENT	RESPONSE / CHANGES MADE
"Well designed and well prepared trial protocol and I would expect good results and will benefit to the severe premature brain injury at last."	Thank you for your kind feedback. We hope the same.

Reviewer 2 (Dr. Dr. Cheryl Battersby) comments:

REVIEWER 1 COMMENT	RESPONSE / CHANGES MADE
"Thank you for asking me to review this protocol of the important ALLO trial, an open-label phase 1 single -arm feasibility and safety study. The ALLO trial aims to assess the feasibility and safety of partially HLA-matched allogeneic UCBC therapy in preterm infants with severe brain injury. Eligible babies are preterm infants born before 28 weeks (ALLO-1) or	Thank you.

between 28 and 36+6 weeks (ALLO-2) gestational age with severe brain injury identified on neuroimaging. Infusion will occur within two to three weeks of confirmation of eligibility, contingent on clinical stability and absence of active sepsis.<br>*Primary outcomes are safety and feasibility.*<br><br>*I have minor recommendations to the main manuscript to improve clarity to the reader , which I appreciate is a shorter and more succinct version of the very comprehensive attached protocol."*	
"1) Abstract: Primary outcome clarity<br>*Suggest to rephrase the primary outcome in the abstract so Primary outcome is clear, rather than in brackets. Change from "Feasibility is defined as having more than 60% of enrolled infants with an eligible allogeneic cord blood unit available .... (primary outcomes)" to "Primary outcome includes: i) feasibility, defined as having more than 60% of enrolled infants with an eligible allogeneic cord blood unit available ii) safety defined as the absence of severe adverse events within 48 hours of infusion or graft-versus-host disease within three months of infusion"*	We have updated this as recommended – thanks for the suggestion.
"2) page 10 "Cell Infusion" duration (one hour) should be included in the intervention section"	We have included this now.
"Abstract<br>*In the "Ethics and Dissemination", rather than "The study complies with the Australian National Statement on Ethical Conduct in Human Research", I would include that there is research approval. "Monash HREC has granted full ethics approval (RES-23-0000-297A), including approval for the research use of allogeneic cord blood from compassionate donations provided by healthy donors, facilitated through the BMDI Cord Blood Bank, which operates as part of the AusCord network. The committee has endorsed the inclusion of patients with*	We have revised this section as suggested.

severe brain injury" or something similar/shorter"	
"Main manuscript:<br>*There is no discussion session. I am not sure if this is a requirement, but I do think some of what is in the main protocol, can come into the discussion, example discussion points to highlight include:*<br>*1) Expanding on the potential immune risk and potential need for HLA matching in this cohort. Need for more explanation on HLA matching (either in intro or discussion).*<br>*2) Justify the 2-3 week period of intervention window with available literature*<br>*3) Justify infusion rate - is there evidence this reduces risk of GVHD? or existing literature to suggest this is optimal rate?*<br>*4) Maybe discuss more about difficulty of risk- benefit analysis and the need for a feasibility and safety study*<br>*5) Also discuss paucity of literature regarding GVHD in neonatal population and the different aims in neonatal cell therapy versus in haematological patients. - that would you expect low levels of GVHD*<br>*6) Also important to add what the treatment is if there is GVHD and the implications short and long term of this condition"*	Thank you for your valuable feedback on the ALLO trial protocol. As BMJ Open protocol papers typically lack a dedicated Discussion section, we initially omitted it. Per your suggestion, we have added a concise Discussion section addressing immune risks, HLA matching, intervention timing, infusion rate, risk-benefit analysis, GVHD literature in neonates, and GVHD management. These additions enhance the manuscript's clarity and scientific rigor while aligning with the journal's format.
"Minor point about terminology: page 15 and throughout. This may be an Australian term, but I wonder if an alternative term can be used to "consumers" for people with lived experience of neonatal care and /or cerebral palsy. It makes it sound commercial or transactional, which it isn't. This may be a term mandated by the Australian authorities but I would consult PPIE groups to make sure this is acceptable, or just say parents or people with lived experience."	We have now revised this term to people with lived experience.
"Page 29 flow chart: some of the fonts are too big and can't see the words in the flow chart"	We have revised the flow chart for better readability.
"Summary:<br>*This is a very important study, and offers*	Thank you for your kind suggestions.

potentially new hope for these infants. I commend the researchers for taking the long protracted, but important and necessary in testing this intervention in these high risk populations. The protocol has been well designed and thought through, and ethically sound. My recommendations were focussing more on readability and suggesting some of the detail of the protocol is brought into a discussion section in the main body of this manuscript. Suggest to accept with minor revisions."	
